# AI-powered automated model construction for patient-specific CFD simulations of aortic flows

**DOI:** 10.1126/sciadv.adw2825

**Published:** 2025-09-05

**Authors:** Pan Du, Delin An, Chaoli Wang, Jian-Xun Wang

**Affiliations:** ^1^ Department of Aerospace and Mechanical Engineering, University of Notre Dame, Notre Dame, IN 46556, USA.; ^2^ Department of Computer Science and Engineering, University of Notre Dame, Notre Dame, IN 46556, USA.; ^3^ Sibley School of Mechanical and Aerospace Engineering, Cornell University, Ithaca, NY 14853, USA.

## Abstract

Image-based modeling is essential for understanding cardiovascular hemodynamics and advancing the diagnosis and treatment of cardiovascular diseases. Constructing patient-specific vascular models remains labor-intensive, error-prone, and time-consuming, limiting their clinical applications. This study introduces a deep-learning framework that automates the creation of simulation-ready vascular models from medical images. The framework integrates a segmentation module for accurate voxel-based vessel delineation with a surface deformation module that performs anatomically consistent and unsupervised surface refinements guided by medical image data. The integrated pipeline addresses key limitations of existing methods, enhancing geometric accuracy and computational efficiency. Evaluated on public datasets, it achieves state-of-the-art segmentation performance while substantially reducing manual effort and processing time. The resulting vascular models exhibit anatomically accurate and visually realistic geometries, effectively capturing both primary vessels and intricate branching patterns. In conclusion, this work advances the scalability and reliability of image-based computational modeling, facilitating broader applications in clinical and research settings.

## Teaser

Automating image-based vascular modeling with deep learning enhances the efficiency and reliability of hemodynamic simulations.

## INTRODUCTION

Cardiovascular disease (CVD) remains one of the leading causes of mortality worldwide, accounting for millions of deaths annually, according to the World Health Organization (*1*). Effectively understanding and managing CVD requires advanced diagnostic tools capable of accurately characterizing complex hemodynamics within the cardiovascular system. Traditional imaging modalities such as computed tomography (CT) and magnetic resonance imaging (MRI) primarily offer high-resolution anatomical detail but typically lack direct measurements of hemodynamic quantities. Advanced imaging techniques such as four-dimensional (4D) flow MRI can capture time-resolved velocity fields and estimate wall shear stress (WSS). Nevertheless, their utility is constrained by limited spatial and temporal resolution, as well as measurement noise. Image-based computational fluid dynamics (CFD) complements these methods by deriving detailed and physics-based hemodynamic information from anatomical images, allowing predictions under conditions that may not be directly measurable by imaging alone.

Moreover, CFD uniquely enables simulations of hemodynamic responses within vascular geometries not yet observed clinically, including those altered by hypothetical surgical interventions or pathological changes. This predictive capability is especially valuable in treatment planning and surgical decision-making, where assessing the potential outcomes of alternative anatomical configurations is critical.

Despite its potential, the clinical application of CFD remains limited. Current CFD methodologies face multiple critical challenges, including geometric inconsistencies resulting from segmentation and smoothing artifacts, simplified modeling assumptions (e.g., rigid vessel walls and Newtonian fluid behavior), and uncertainties in boundary conditions such as prescribed inflow profiles, Windkessel parameters, and vessel wall elasticity. Among these challenges, efficient and accurate construction of patient-specific models remains one of the most critical barriers to clinical translation (*2*–*4*).

Constructing patient-specific vascular models for image-based CFD involves multiple steps, including image segmentation, geometry modeling, and mesh generation for the computational domain, all of which are critical to ensuring the fidelity of the final simulation results. However, the standard workflow heavily relies on manual methods, making it highly labor-intensive and time-consuming. For instance, in SimVascular—a widely used cardiovascular simulation platform (*5*)—experts delineate the centerline of blood vessels, segment the lumen boundary across individual 2D slices perpendicular to the centerline, and reconstruct the vascular geometry using B-splines. This process demands meticulous effort and often takes hours or even days to complete for a single patient, making it impractical for real-time or high-throughput applications (*6*, *7*). Furthermore, the reliance on operator expertise introduces considerable subjectivity, leading to variability between cases and potential errors such as inaccurate branch junctions and boundary delineation (see more analysis in Supplementary Text) (*8*). These issues are further exacerbated by variations in image quality and acquisition protocols, which make it difficult to achieve reproducible and accurate geometries (*9*). Together, these limitations undermine the reliability of simulations and pose major barriers to the integration of image-based modeling into clinical workflows.

Automating the process of constructing patient-specific vascular models is essential to overcome the limitations of manual workflows, particularly in terms of efficiency, consistency, and scalability. A fully automated pipeline can minimize operator dependency, reduce variability, and enable rapid generation of reproducible geometries, making image-based computational modeling more practical for clinical applications (*10*). Early attempts to automate this workflow primarily relied on traditional methods, such as deformable contour models (DCMs) (*11*–*17*), active surface methods (ASMs) (*18*–*23*), and level set methods (*24*–*28*). These techniques introduced some level of automation by optimizing geometric models to fit image data based on predefined energy functions. For example, DCMs minimized energy terms composed of internal forces promoting smoothness and external forces aligning the contour with image gradients (*11*, *13*). ASMs extended DCMs to 3D scenarios, enhancing their utility for complex structures. While effective in certain cases, these methods often struggled with complex vascular geometries and imaging noises. They also required precise initialization and parameter tuning, undermining their robustness and practicality in nontrivial scenarios (*22*, *23*). The level-set method offered another mathematical approach to segmentation by evolving a scalar field over time to extract zero-level isosurfaces that represent the boundaries of target vascular geometries (*24*, *25*). This evolution was guided by image features, such as gradients or region-based information, to delineate vascular structures more accurately. These methods achieved success in segmenting large vessels/ventricles and were incorporated into open-source platforms such as the Vascular Modeling Toolkit (*24*). However, they also remained highly sensitive to initialization, imaging noise, and hyperparameters, often requiring extensive case-specific fine-tuning and user intervention. Notably, level-set methods tended to produce false detections, particularly in images with low peak signal-to-noise ratios (PSNRs) or serious artifacts. Their reliance on threshold-based parameterization further limited their generalizability across diverse cases (*27*, *28*).

The introduction of deep learning (DL) has substantially advanced medical image segmentation by offering data-driven solutions that outperform traditional methods in terms of accuracy and robustness (*29*, *30*). DL-based segmentation models, particularly those using convolutional neural networks (CNNs) and encoder-decoder architectures, leverage large-annotated datasets to directly learn the mapping between image inputs and semantic segmentation outputs. These methods have demonstrated remarkable success in segmenting cardiovascular structures, including ventricles, arteries, and capillaries (*31*–*36*). However, a critical limitation of most DL-based segmentation approaches is their focus on identifying the region of interest using binary voxel classifications, without facilitating the creation of 3D computational meshes that can be used for downstream CFD or fluid-structure interaction (FSI) simulations. To use the voxel-based outputs for computational modeling, additional postprocessing steps, such as marching cubes (*37*), are required to generate smooth surfaces. This process can introduce artifacts, reduce geometric fidelity, and undermine the accuracy of simulations. To address these challenges, recent studies have explored integrated frameworks that combine voxel segmentation and surface reconstruction. For example, deep active surface models (DASMs) use graph CNNs (GNNs) to iteratively deform surfaces by simultaneously minimizing energy losses and surface regularization terms (*38*, *39*). Similarly, methods such as Voxel2Mesh integrate voxel-based segmentation with surface deformation using encoder-decoder networks for voxel predictions and GNNs to represent and deform surface mesh (*40*–*42*). In these methods, features extracted from the encoder are projected onto the surface vertices and integrated into the hidden layers of the GNN to guide deformations. Despite their promise, these approaches face challenges when dealing with multibranched vascular geometries, as they require handling severe nonrigid transformations. Nonrigid deformation has been extensively studied in the computer vision community, where various DL-based shape registration algorithms have shown impressive performance in aligning complex geometries (*43*–*46*). Some of these methods incorporate physics-based deformation models, such as large deformation diffeomorphic metric mapping (LDDMM) (*47*), to ensure smooth transformations. For instance, Amor *et al.* (*45*) combined a ResNet structure with LDDMM to align geometries from datasets that included human organs such as the heart and ventricles. However, despite their success in broader shape registration problems, these methods have yet to see extensive application in surface reconstruction for cardiac image segmentation.

Another critical limitation of most DL-based segmentation models is their reliance on manually crafted surface labels, which are often assumed to be the gold standard despite inherent imperfections. For instance, in SimVascular, vascular geometries are reconstructed using nonuniform rational B-splines (NURBS) fitted to discrete slices along manually identified vessel centerlines (*5*). While this approach provides an approximation of the vascular surface, most of the surface points do not directly correspond to image-derived features, leading to inaccuracies. In addition, SimVascular uses blending operations to smooth junctions between vessels (*48*), introducing artifacts that might be inconsistent with the image gradients. These imperfections in training labels propagate through segmentation pipelines, limiting the accuracy and generalizability of supervised DL models.

Despite these challenges, integrating DL-based segmentation with robust surface reconstruction frameworks provides a promising pathway to enhance the automation and accuracy of image-based cardiovascular modeling. In this work, we propose a novel DL framework that unifies voxel segmentation and surface deformation into a single, cohesive pipeline for the automated construction of patient-specific models for image-based CFD simulations of aortic flows.

The proposed framework begins with a voxel-based segmentation module leveraging a novel Laplacian-of-Gaussian–based Bayesian network (LoGB-Net) (*49*). Specifically designed to enhance segmentation accuracy across vascular structures of varying scales, LoGB-Net incorporates Bayesian inference principles, providing robustness to image noise and enabling uncertainty quantification (UQ). This capability is particularly advantageous for identifying subtle or ambiguous vessel boundaries frequently encountered in medical imaging. The module produces a high-resolution binary voxel map, serving as the foundational input for subsequent surface reconstruction.

To transform the voxel-based segmentation into anatomically accurate surface models, we developed a surface deformation model that integrates the LDDMM algorithm with GNNs, enabling the framework to effectively capture complex, nonrigid transformations while inherently enforcing smooth and physically plausible surface deformations. Notably, our approach eliminates the need for traditional surface regularization terms, as smoothness and anatomical fidelity are implicitly ensured by the LDDMM formulation. The deformation process is guided by an unsupervised learning objective comprising an energy term that aligns reconstructed surfaces with underlying image gradients to ensure geometric consistency, along with additional constraints preventing unrealistic local deformations.

Overall, the framework strategically combines supervised and unsupervised learning paradigms. The LoGB-Net module operates in a supervised setting, leveraging labeled voxel data exclusively during the training phase to effectively adapt to diverse imaging conditions and accurately delineate vascular structures. On the other hand, the surface deformation module uses unsupervised learning, using alignment losses and image gradient–based energy terms without requiring manually annotated surface labels. For benchmarking and validation purposes only, manually constructed reference models generated using SimVascular are used. Complete details regarding training/test sets are provided in the “Dataset creation and preprocessing” section.

**Fig. 1.**
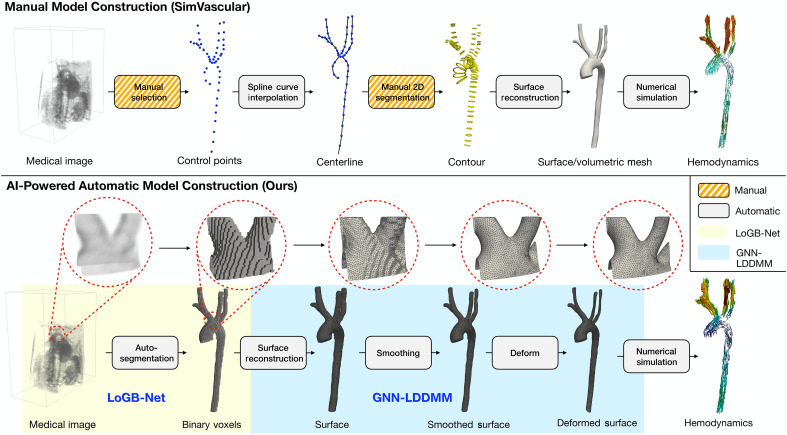
Comparison of manual and AI-powered automatic model construction workflows for image-based simulations. Representative results at each step of our proposed framework are shown in the bottom panel.

Figure 1 provides a schematic overview comparing our proposed automated framework against the traditional manual workflow in SimVascular (*5*) using a representative test sample. The top panel illustrates the conventional manual workflow, involving manual selection of vessel centerlines, spline interpolation, and manual segmentation of multiple 2D image slices for vascular surface reconstruction. Although capable of producing high-quality models, this approach remains labor-intensive, highly time-consuming, and prone to operator-induced variability.

In contrast, the bottom panel of Fig. 1 illustrates our automated approach. The LoGB-Net module generates a binary voxel segmentation accurately capturing the overall vascular shape, albeit with discrete, voxelized characteristics inherent to the representation. Subsequently, the GNN-LDDMM module refines this coarse segmentation by smoothly deforming it into an anatomically realistic surface mesh. The final reconstructed surface closely matches underlying anatomical details, resulting in geometries directly suitable for CFD simulations. Our approach greatly reduces processing time while preserving anatomical accuracy and reproducibility, marking a substantial advancement in patient-specific cardiovascular modeling.

The following sections systematically evaluate the proposed framework’s performance in its two core components: voxel-based segmentation and surface deformation. First, we quantitatively assess LoGB-Net’s segmentation accuracy against several established machine learning baselines, emphasizing its enhanced vascular delineation capabilities and unique UQ features. Next, we rigorously evaluate the surface deformation module by comparing reconstructed geometries with manually crafted models from SimVascular.

## RESULTS

### Voxel segmentation evaluation

The binary voxel segmentation performance of the proposed LoGB-Net model was evaluated against 10 state-of-the-art (SOTA) DL-based segmentation methods, including FPN (*50*), U-Net 3D (*51*), PSPNet (*52*), nnUNet (*53*), Attention-UNet (*54*), MISSFormer (*55*), Swin-UNET (*56*), TransUNet (*57*), UNEt TRansformers (UNETR) (*58*), and UNETR++ (*59*). The proposed LoGB-Net features a “shape stream” module specifically designed to detect vessels of varying diameters, which is critical for accurately modeling multibranch aortic geometries and other tubular cardiovascular structures, such as cerebral and pulmonary arteries. More details can be found in the “LoGB-Net: A multiscale vascular segmentation model” section. Among the baseline methods, UNETR uses residual blocks to improve feature extraction, while Attention-UNet uses attention mechanisms to focus on critical regions selectively. These SOTA methods, along with the others evaluated, represent the current advances in 3D medical image segmentation and provide a robust benchmark for assessing LoGB-Net’s performance.

**Fig. 2.**
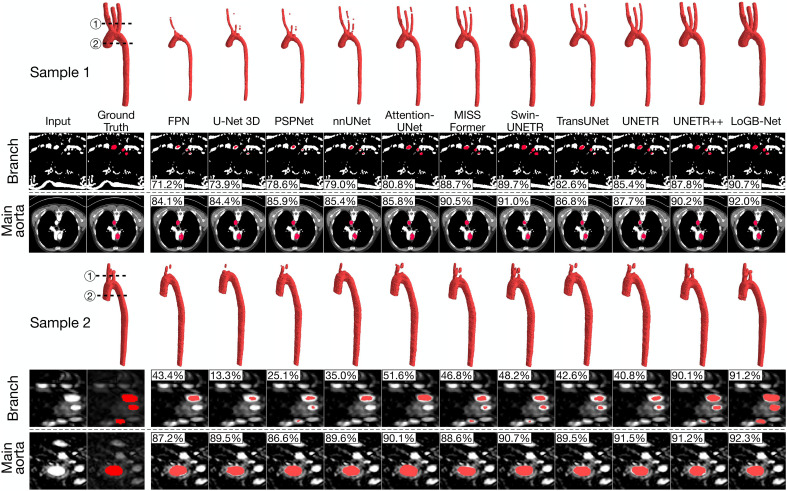
Comparison of segmentation results for two representative samples across different models. The top row for each sample shows 3D reconstructions of segmented aortic geometries, while the bottom rows provide cross-sectional views at the supra-aortic branches (branch) and the main aorta (main aorta). LoGB-Net demonstrates superior performance, producing anatomically accurate and continuous segmentations, particularly in challenging regions such as the supra-aortic branches.

Figure 2 compares segmentation results across models for two representative samples, and metrics—including dice similarity coefficient (Dice), average surface distance (ASD), and Hausdorff Distance—are computed for quantitative assessment. For both samples, the top row shows 3D reconstructions of segmented vascular structures, while the bottom rows provide cross-sectional views at two critical locations: the supra-aortic branches (branch) and the main aorta (main aorta), accompanied by 2D Dice scores for each cross-sectional plane. While most models perform well in segmenting the main aorta, the substantial variability is observed in segmenting smaller, more complex branches. Models such as FPN, U-Net 3D, PSPNet, and nnUNet frequently produce incomplete or disconnected segmentations for the upper branches, such as the right and left common carotid arteries and the subclavian arteries. This is quantitatively supported by the 2D Dice scores, where these models score substantially lower for branch views, ranging from 71.2 to 79.0% in sample 1 and from 13.3 to 43.4% in sample 2. Attention-based models—including Attention-UNet, MISSFormer, Swin-UNETR, and TransUNet—improve continuity but still fail to consistently achieve anatomically accurate results, particularly for challenging regions in test sample 2. Statistically, the Dice score ranges for branches increase to 80.8 to 89.7% and 42.6 to 51.6% for samples 1 and 2, respectively, reflecting a notably improved yet still suboptimal performance. By contrast, UNETR++ and our LoGB-Net demonstrate superior performance, with LoGB-Net consistently producing the most accurate and continuous segmentations, yielding Dice scores exceeding 90% across all cross-sectional views. The inclusion of the LoGB stream module enhances its ability to detect vessels across a wide range of diameters, contributing to its improved performance in segmenting smaller and more complex structures.

Table 1 provides quantitative results for all testing samples, reinforcing the visual findings. LoGB-Net achieves the highest Dice coefficients (92.7% for branch vessels and 93.7% for the main aorta), the lowest ASD values (0.678 mm for branch vessels and 0.682 mm for the main aorta), and competitive Hausdorff distances for both vessel types. UNETR++ shows comparable overall performance: It achieves a slightly better Hausdorff distance (6.03 mm) than LoGB-Net (6.23 mm) for branch vessels but lower Dice scores (main vessels: 91.2 versus 93.7; branch vessels: 89.4 versus 92.7) and slightly higher ASD (main vessels: 0.74 mm versus 0.68 mm).

**Table 1.** Quantitative results of different methods. Each value is reported as means ± SD across five runs. The table compares CNN-based, attention-based, and our method for segmentation performance on the aortic branch and main regions. Bold denotes best performance. ReLU, rectified linearunit.

Region	Metric	FPN	U-Net3D	PSPNet	nnUNet	Att-UNet	MISSFormer	SwinUNETR	TransUNet	UNETR	UNETR++	LoGB-Net (Ours)
Branch	Dice ↑	0.72 ± 0.02	0.76 ± 0.04	0.78 ± 0.04	0.77 ± 0.07	0.82 ± 0.03	0.88 ± 0.02	0.90 ± 0.02	0.82 ± 0.02	0.88 ± 0.03	0.89 ± 0.04	**0.93** ± **0.01**
	ASD ↓	1.52 ± 0.32	1.38 ± 0.31	1.33 ± 0.30	1.21 ± 0.32	0.93 ± 0.21	0.81 ± 0.20	0.79 ± 0.25	0.93 ± 0.28	0.70 ± 0.17	0.68 ± 0.16	**0.68** ± **0.17**
	Hausdorff ↓	8.49 ± 3.25	8.32 ± 4.02	9.77 ± 3.77	8.94 ± 2.55	6.73 ± 0.29	6.90 ± 0.08	6.32 ± 0.13	6.45 ± 0.24	6.14 ± 0.70	**6.03** ± **0.36**	6.23 ± 0.96
Main	Dice ↑	0.73 ± 0.02	0.77 ± 0.03	0.78 ± 0.03	0.78 ± 0.03	0.85 ± 0.02	0.90 ± 0.02	0.91 ± 0.03	0.90 ± 0.02	0.90 ± 0.04	0.91 ± 0.02	**0.94** ± **0.01**
	ASD ↓	1.45 ± 0.36	1.34 ± 0.34	1.34 ± 0.31	1.40 ± 0.31	0.88 ± 0.23	0.92 ± 0.23	0.80 ± 0.22	0.93 ± 0.31	0.69 ± 0.20	0.74 ± 0.19	**0.68** ± **0.20**
	Hausdorff ↓	10.25 ± 3.78	11.56 ± 3.93	9.99 ± 4.20	9.29 ± 2.33	6.94 ± 0.31	**6.13** ± **0.19**	7.02 ± 0.07	6.88 ± 0.25	6.42 ± 0.45	6.32 ± 0.24	6.32 ± 1.49

In addition to the fully automatic segmentation approaches presented here, semi-automatic methods such as the SeqSeg model by Sveinsson *et al.* (*60*) have demonstrated comparable SOTA performance. Although requiring manual input, SeqSeg exhibits excellent generalization. A detailed comparison with SeqSeg is included in Supplementary Text.

### Surface construction analysis

After obtaining the segmented voxel image, we construct the surface and fine-tune it for better alignment to the image gradient. This section presents a detailed comparison of the resulting surfaces and corresponding CFD solutions between our deformed surface and manual ones derived from open-source, expert-verified datasets.

**Fig. 3.**
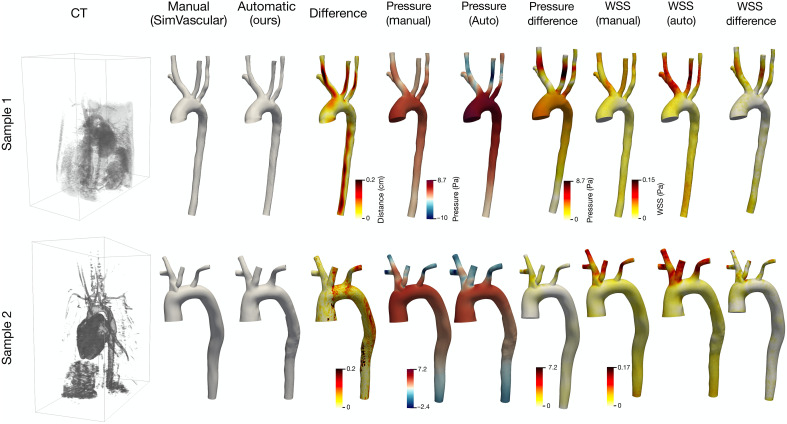
Comparison of manual and automated surface reconstruction for two representative samples. Columns display the input CT image, manual and automated reconstructed surfaces, geometric difference maps, and the resulting pressure and WSS distributions from CFD simulations.

As shown in Figure 3, both manual (surface labels created by experts using SimVascular) and automated (ours) methods successfully reconstruct vascular geometries, capturing the essential regions within the CT dataset. On visual inspection, the reconstructed surfaces appear highly similar, with the automated method matching the global geometric features produced by manual reconstruction. A detailed difference map, shown as the distance contour, highlights some subtle geometric discrepancies, which are predominantly localized in branch regions and areas with more complex vessel intersections. These differences are attributed to the inherent smoothing of manual methods and the comprehensive point-wise optimization in our approach, which ensures tighter adherence to image features. We also computed CFD results, including pressure and WSS, on these meshes and displayed them on the right to demonstrate the downstream prediction of hemodynamic information. Specifically, the maximum pressure values on the manual surface are 5.09 and 6.25 Pa for samples 1 and 2, respectively, lower than those predicted on the automated surface (8.61 and 7.16 Pa). Conversely, the automated surfaces show more negative minimum pressures (−11.89 Pa and −2.43 Pa) compared to the manual ones (−1.62 and −2.19 Pa), indicating a broader pressure variation captured by the automated reconstruction. Similarly, for WSS, the automated surface yields higher maximum values (0.15 and 0.17 Pa) compared to the manual reconstructions (0.08 and 0.16 Pa). To further quantify these differences, we computed the mean and SD of the absolute pressure and WSS differences. For pressure, the mean and SD were 2.47 and 1.55 Pa for sample 1, and 0.40 Pa and 0.45 Pa for sample 2. For WSS, the corresponding values were 0.014 Pa and 0.016 Pa for sample 1 and 0.002 and 0.014 Pa for sample 2. According to the difference contour maps, larger differences are primarily localized in the upper branches, vessel junctions, and the ascending aorta.

**Fig. 4.**
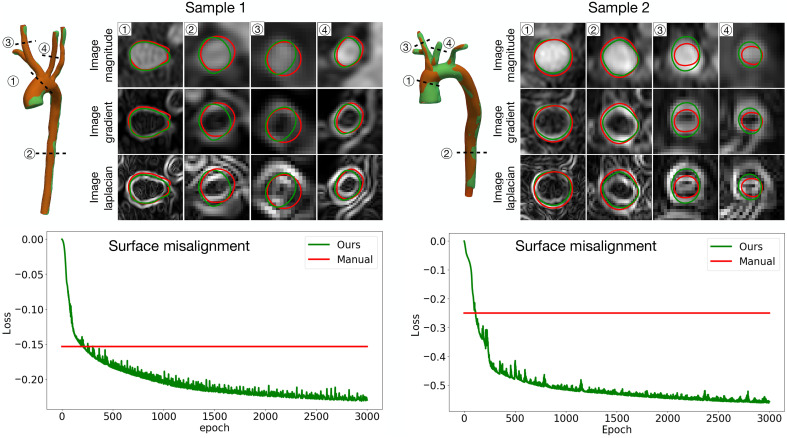
Detailed cross-sectional comparison of reconstructed surfaces and source images for two representative samples. For each cross section, overlays of the manual surface (red) and the automated surface (green) are shown against three background references: image magnitude, image gradient, and image Laplacian. The bottom panels present the surface misalignment loss during training, where the automated surfaces achieve rapid convergence to lower loss values compared to static, manual surfaces.

Figure 4 provides a detailed cross-sectional comparison between manual and automated surfaces at four representative locations (labeled 1 to 4) for two samples. For each cross section, overlays of the surfaces are shown against three background images: image magnitude, image gradient, and image Laplacian. In the magnitude images, the automated surface (green) consistently aligns slightly better with the vessel boundaries, while the manual surface (red) often deviates due to oversmoothing. For example, in section 1 of sample 1, the manual surface fails to fully capture the narrow region of the vessel wall, leading to an underrepresentation of the actual geometry. Similarly, in section 4 of sample 2, the manual surface erroneously extends into adjacent regions, an artifact caused by interpolation errors in the manual workflow. The gradient images emphasize regions of high-intensity change, which correspond to vessel boundaries. Here, the automated surface matches closely to the middle of the high-gradient regions, as seen in sections 2 and 3 of both samples. By contrast, the manual surface slightly deviates, particularly in high-curvature or branching areas, such as section 3 of sample 2, where it cuts into the vessel interior. Laplacian images, which highlight transitions between regions of different intensities, can further show the alignment between reconstructed surfaces and images.

The lower panels in Fig. 4 quantitatively evaluate surface alignment through the energy loss term, which measures misalignment between the reconstructed surface and the image gradients during the deformation process. For both samples, the automated pipeline rapidly reduces the loss over the course of training and converges to substantially lower values compared to the manually reconstructed surfaces. Notably, the manual surfaces exhibit a static loss value, reflecting their inability to adjust beyond the initial annotations. In contrast, the automated surfaces continuously optimize their alignment throughout the unsupervised optimization process, achieving superior adherence to image features by approximately the 200th epoch. To quantify the improvement in surface alignment, we computed the surface alignment energy as the logarithmic negative surface misalignment error and compared it to the manual reference. This analysis showed improvements of 9.4 and 34.9% in surface alignment for samples 1 and 2, respectively. These results demonstrate that our pipeline not only automates surface reconstruction, substantially reducing the labor-intensive nature of manual workflows, but also delivers geometries with enhanced fidelity to the source images.

### Uncertainty estimation and propagation

**Fig. 5.**
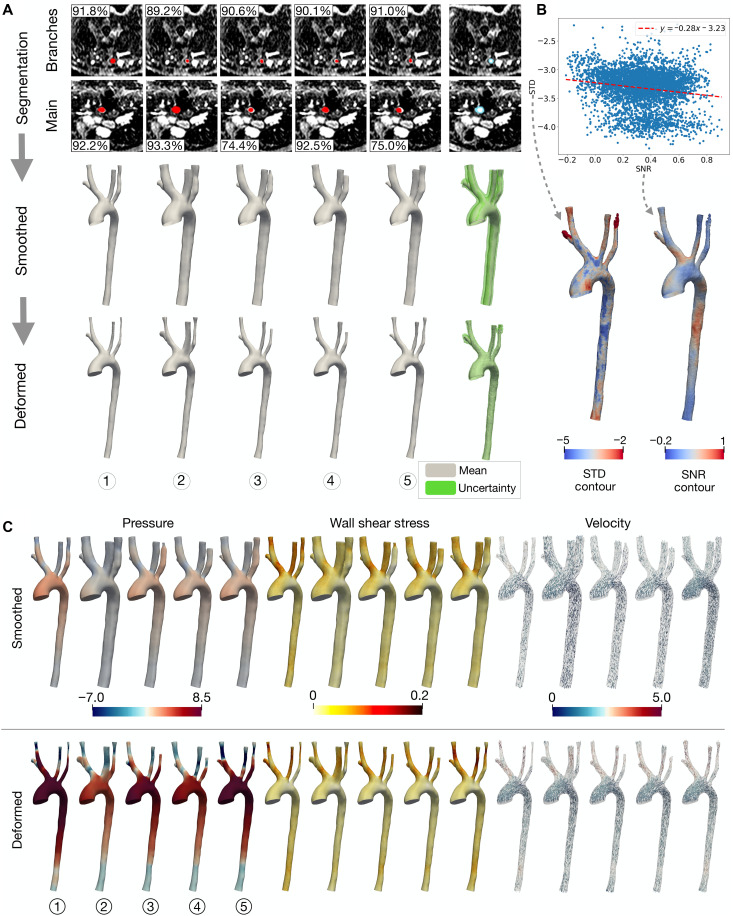
Uncertainty estimation and propagation through surface reconstruction and CFD simulation. (**A**) Segmentation uncertainty visualized across voxel-based predictions, smoothed geometries, and deformed surfaces, with uncertainty maps highlighting regions of variability. (**B**) Correlation between uncertainty (SD) and image quality (SNR), with corresponding surface contours. (**C**) Comparison of hemodynamic quantities from CFD simulations for smoothed and deformed surfaces.

This section explores the UQ capabilities of the LoGB-Net model and demonstrates how uncertainties propagate through the GNN-LDDMM surface deformation and CFD simulation steps. Figure 5A showcases random segmentation realizations obtained by sampling the learned probabilistic distributions of the model parameters of the LoGB-Net. The top row illustrates five representative voxel-based segmentation results for both the main aorta and the branches, with the rightmost column depicting uncertainty regions that highlight areas of potential edge variability. These uncertainty estimates are crucial for understanding the confidence levels of the segmentation outputs. As shown by the metrics in the figure, LoGB-Net exhibits up to 20% variability in Dice scores across the sampled segmentation realizations for the presented cross sections.

The second row presents the smoothed geometries derived directly from the voxel-based outputs using a standard smoothing algorithm to produce simulation-read meshes. The uncertainties from the segmentation step persist, particularly near complex vessel regions (see green bands in the rightmost column). The bottom row demonstrates the results after applying the GNN-LDDMM deformation module, which aligns the surface geometry with underlying medical image gradients. Compared to the smoothed geometries, the deformed results exhibit markedly reduced uncertainty, particularly in the main aorta, as seen in the tighter green bands around the mean surface.

Figure 5B analyzes the relationship between segmentation uncertainty and image quality. The scatter plot correlates the log of the SD of uncertainty with the SNR across all points on the mean surface. A negative correlation is observed, with a regression slope of −0.28, indicating that regions with higher SNR exhibit lower uncertainty. This trend is further supported by the SD and SNR contour plots overlaid on the mean surface. Regions with high uncertainty, such as the distal ends of branch vessels, correspond to areas of lower SNR, highlighting the impact of poor image quality on segmentation reliability.

Figure 5C compares CFD solutions for pressure, WSS, and velocity fields across the smoothed and deformed surface ensembles. The results reveal substantial differences in hemodynamic outcomes, even for minor geometric discrepancies. Deformed surfaces yield higher average pressure and WSS values compared to smoothed ones, reflecting the improved anatomical alignment achieved by the deformation module. While the main aorta geometries converge closely after deformation, branch geometries still exhibit notable variations due to persistent noise in these regions.

Overall, our framework leverages UQ to generate ensembles of potential segmentation outcomes, moving beyond single-point predictions and offering a probabilistic understanding of geometric variability. The GNN-LDDMM deformation module plays a crucial role in refining these surfaces, reducing uncertainty, and improving alignment with medical images.

### Framework ablation study

#### Ablation study of the segmentation module

To evaluate the necessity and effectiveness of the key components in the LoGB-Net segmentation model, we conducted a comprehensive ablation study focusing on the LoG stream, Bayesian framework, and balanced gate (see Table 2). The segmentation performance was assessed under various configurations, with each component removed individually. The results in the left three columns demonstrate substantial performance degradation when any of these components is excluded. This finding demonstrates the unique contribution of each subcomponent in the overall architecture. The LoG stream, serving as the core feature of LoGB-Net, has the most pronounced impact on performance. Its multiscale kernel setup enhances the model’s ability to detect vessels of varying diameters, making it indispensable for handling complex vascular geometries. The balanced gate addresses foreground-background imbalance during training, ensuring stable and robust optimization. Meanwhile, the Bayesian framework improves robustness by learning the probability distribution of kernel parameters, which captures the inherent variability in medical image data.

**Table 2.** Quantitative results of the LoGB-Net’s ablation study. $L_{\text{LoG}}^{-}$ , $L_{\text{Bay}}^{-}$ , and $L_{\text{Gate}}^{-}$ represent LoGB-Net without LoG module, Bayesian optimization, and balanced gate, respectively. L(1) , L(2) , L(3) , L(4) , and L(5) represent LoGB-Net with 1, 2, 3, 4, and 5 LoG layers, respectively. Bold denotes best performance.

	Metric	$L_{\text{LoG}}^{-}$ []L(0)	$L_{\text{Bay}}^{-}$	$L_{\text{Gate}}^{-}$	L(1)	L(2)	L(3)	L(4)	L(5) (ours)
SA	Dice ↑	0.735 ± 0.033	0.863 ± 0.031	0.896 ± 0.012	0.852 ± 0.033	0.863 ± 0.018	0.872 ± 0.011	0.893 ± 0.033	**0.927** ± **0.011**
	ASD ↓	1.384 ± 0.334	0.810 ± 0.214	0.801 ± 0.179	1.291 ± 0.123	1.226 ± 0.202	0.922 ± 0.244	0.772 ± 0.202	**0.678** ± **0.168**
	Hausdorff ↓	8.271 ± 2.276	7.993 ± 2.112	7.909 ± 1.093	8.203 ± 1.903	8.014 ± 1.221	7.027 ± 0.721	6.626 ± 0.121	**6.225** ± **0.957**
SA	Dice ↑	0.741 ± 0.022	0.871 ± 0.033	0.882 ± 0.021	0.866 ± 0.014	0.865 ± 0.009	0.877 ± 0.036	0.923 ± 0.003	**0.937** ± **0.006**
	ASD ↓	1.399 ± 0.123	0.961 ± 0.120	0.933 ± 0.200	1.105 ± 0.239	0.979 ± 0.103	0.933 ± 0.210	0.727 ± 0.121	**0.682** ± **0.197**
	Hausdorff ↓	8.335 ± 2.996	8.220 ± 2.003	8.011 ± 1.898	8.277 ± 2.516	8.100 ± 2.274	7.965 ± 1.001	7.482 ± 1.112	**6.322** ± **1.485**

To further understand the role of the LoG stream, we experimented with varying the number of LoG layers, represented as L(1) to L(5) in Table 2. The results show a clear trend: As the number of layers increases, performance improves across all metrics. This demonstrates that deeper hierarchical structures provide more effective multiscale vessel detection. However, performance appears to plateau at L(5) , suggesting that additional layers may yield diminishing returns in this context.

#### Ablation study of the GNN-LDDMM deformation module

**Fig. 6.**
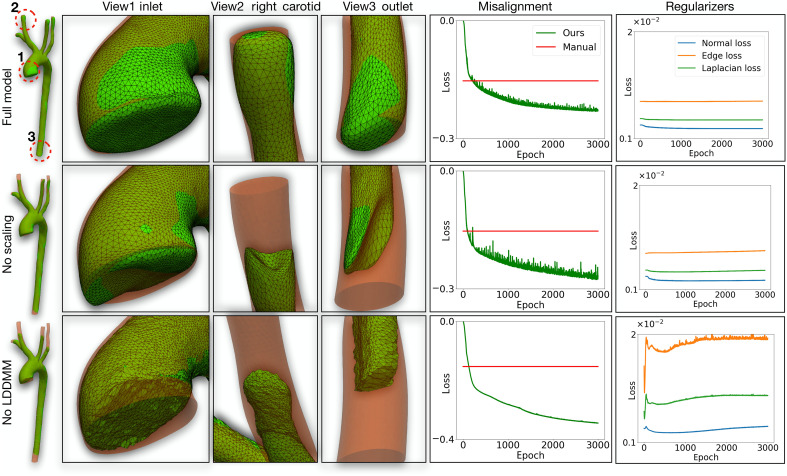
Ablation study for deformation model. Mesh comparison in different views (first to fourth columns) and loss curve (fifth column).

To evaluate the necessity and effectiveness of key components within the GNN-LDDMM deformation model, we conducted an ablation study, as illustrated in Fig. 6. Specifically, we examined the impact of removing critical elements such as the LDDMM module and the scaling component. These investigations provide insight into the importance of these elements in ensuring geometrically accurate and physically plausible surface deformations for subsequent CFD analysis.

The left four columns of Fig. 6 display surface visualizations under different ablation scenarios. The absence of the scaling component leads to retraction of inlets and outlets, causing unrealistic geometries. This occurs because image gradients alone do not sufficiently constrain surface points in these areas, allowing them to drift toward regions with locally lower misalignment energy. The scaling component addresses this by stabilizing these regions, preserving vessel lengths and anatomical consistency. Furthermore, removing the LDDMM module results in substantial surface faults, particularly at the inlet and outlet regions. This deficiency arises because LDDMM acts as a strong prior for maintaining surface continuity and anatomical realism, which is crucial for ensuring the feasibility of downstream CFD routines. Without LDDMM, the deformation process fails to preserve surface smoothness and structural integrity. In contrast, the full model incorporating both LDDMM and scaling components (first row) produces smooth, realistic vascular surfaces that align well with underlying medical images.

Quantitative evaluations further reinforce the visual observations. The rightmost two columns of Fig. 6 illustrate the progression of misalignment losses and surface smoothness indicators across training epochs. In all configurations, the misalignment losses are relatively low, indicating overall good alignment with the underlying medical image data. Both the “no LDDMM” and “no scaling” cases exhibit lower misalignment losses compared to the full model. However, this reduction comes at a substantial cost to geometric integrity, as evidenced by increased surface irregularities and unrealistic deformations in critical regions. These findings demonstrate that the optimization process tends to prioritize minimizing energy loss over preserving surface quality when structural constraints are absent. The inclusion of both LDDMM and scaling components is, therefore, essential for enforcing geometric fidelity and ensuring anatomically realistic surface reconstructions, balancing alignment accuracy with physical plausibility. This is also critical for obtaining reliable downstream CFD simulation results, as geometric variations can substantially affect flow predictions. Additional comparison results are provided in Supplementary Text.

## DISCUSSION

### Effectiveness of the proposed LoGB-Net and GNN-LDDMM deformation module

The proposed framework streamlines the image-to-voxel-to-mesh pipeline, achieving full automation in a single pass. The comprehensive numerical results demonstrate its superior segmentation accuracy and mesh construction quality, validated by quantitative metrics, visual comparisons, and CFD simulations. In particular, Fig. 2 shows that LoGB-Net stands out among existing SOTA methods (with performance closely matched by UNETR++), exhibiting strong capability to reliably segment complex, multibranch vascular geometries with minimal manual intervention. Figure 3 further compares the surface generated by our automated method with a manually constructed surface from SimVascular. Another noteworthy observation is that the two samples presented in Fig. 3 exhibit different supra-aortic branching topologies: Sample 1 is from a human aorta where the LCCA branches directly from the aortic arch, whereas sample 2 is from a rabbit aorta in which the LCCA arises from the brachiocephalic trunk. Our model successfully handled both branching configurations, demonstrating its ability to generalize across topological variations

As discussed above, the most notable advantage of the proposed framework is its ability to generate simulation-ready vascular geometries with minimal human intervention. Traditional manual workflows, such as those used in tools such as SimVascular, are labor-intensive, time-consuming, and highly dependent on operator expertise. These manual approaches often involve intricate tasks such as centerline extraction, cross-sectional segmentation, and surface interpolation, each prone to variability and subjective interpretation. In contrast, the proposed framework automates the whole process while ensuring anatomical accuracy. The surface deformation module further improves geometric fidelity by aligning reconstructed surfaces with underlying medical image gradients, eliminating artifacts commonly introduced by manual methods, such as oversmoothing or interpolation errors at junction regions.

While manual and automated geometries may appear visually similar, the CFD analysis results (Fig. 2) show that even minor geometric discrepancies can lead to substantial differences in simulated hemodynamic features such as pressure, WSS, and velocity distributions. These parameters are critical biomarkers for CVD diagnosis and treatment planning. Prediction errors in these biomarkers could substantially influence clinical decision-making. However, without clinical ground truth for flow fields, it is inherently difficult to determine the absolute accuracy of CFD predictions, making definitive validation of simulation results challenging. Consequently, quantifying geometric uncertainty becomes critical to ensure the reliability of CFD-based analyses. The proposed framework incorporates UQ to assess segmentation variability and its propagation through surface deformation and CFD simulations. This capability enables the generation of an ensemble of plausible geometries and flow solutions, allowing users to better understand the range of potential outcomes. Our results demonstrate that uncertainty is substantially reduced in regions such as the main aorta through the image-guided surface deformation process, reflecting the robustness of the framework in achieving consistent and reliable results. However, persistent uncertainties in distal branches and areas with poor image quality highlight the influence of imaging limitations on segmentation performance.

### Limitation and future prospects of current framework

Despite these strengths, the study has certain limitations that warrant further investigation. One limitation is the potential challenge faced by the LDDMM module when handling large deformations, such as those arising from poor initial segmentation results. In these cases, the deformation model may struggle to achieve accurate geometric reconstructions, particularly in highly distorted or irregular regions. This limitation underscores the need for more robust deformation methods or hybrid approaches to handle cases with substantial discrepancies between the segmented geometry and the underlying image. Another limitation is the relatively small and homogeneous training dataset used to develop and evaluate the framework, primarily due to the limited availability of openly accessible, patient-specific image datasets with corresponding voxel and surface labels. This constraint is the main factor limiting our model’s ability to generalize to more complex anatomical variations. The model itself, however, is inherently topology-agnostic: Both the segmentation module (LoGB-Net) and the deformation module (GNN-LDDMM) operate without relying on predefined centerlines, tree templates, or fixed connectivity assumptions.

To maximize robustness and generalizability, we made every effort to collect diverse aortic samples, and we were fortunate to include a degree of anatomical variety: Our training set contains both human and rabbit aortas (with differing supra-aortic branching topologies) as well as multiple aortic regions, including thoracic, abdominal, and iliac bifurcations (sourced from the multicenter aortic vessel (AVT) dataset). As a result, our model successfully handles varying supra-aortic branching patterns (discussed in the “Effectiveness of the proposed LoGB-Net and GNN-LDDMM deformation” section) and accurately detects abdominal and iliac structures when tested on previously unseen AVT cases. Additional evidence is provided in Supplementary Text, where our results are compared with the SOTA semi-automatic SeqSeg method. Despite this demonstrated level of generalizability, the ultimate limitation remains the scope of available training data. Incorporating more patient-specific cases with rare or complex anatomies—such as atypical supra-aortic branching patterns, congenital anomalies, or pathological variations such as dissection—will be essential to further enhance the model’s capacity to accurately segment a broader spectrum of aortic anatomies with high clinical utility.

In addition, the limited size and quality of the training dataset contribute to the overall lower segmentation performance for the baseline models. For instance, our nnUNet baseline achieves lower accuracy (Dice = 0.780) compared to the results reported in the SeqSeg study (Dice = 0.889). SeqSeg used centerline-guided patch extraction, ensuring vessel presence within every training patch. In contrast, our random patch-sampling strategy across the entire image volume includes substantial amounts of non-aortic tissues and backgrounds, increasing task complexity but enhancing the model’s robustness to irrelevant regions.

Furthermore, the robustness of LoGB-Net and GNN-LDDMM to severe image noise or poor-quality imaging data remains limited. For instance, distal branches and regions with low SNR often exhibit higher errors and poor robustness, which can propagate to CFD simulations (shown in Fig. 5). This variability highlights a limitation of the current deformation model in handling severe noise, which is often encountered in real-world imaging datasets. Enhancing the resilience of the framework to these challenges may involve additional anatomical priors, advanced noise-reduction techniques, or improved regularization strategies during the deformation process.

### Broader implications

The automated and robust nature of the proposed framework has important implications for advancing patient-specific cardiovascular modeling. By reducing the time, labor, and variability associated with manual workflows, this framework paves the way for integrating CFD-based analyses into routine clinical workflows. The incorporation of UQ further enhances the reliability of the results, enabling clinicians to make more informed decisions regarding diagnosis and treatment planning. Furthermore, the modular design of the framework allows for its extension to other anatomical regions and medical imaging modalities, broadening its applicability across various domains of computational medicine.

In conclusion, this study demonstrates the potential of combining DL with image-driven optimization to transform the field of patient-specific vascular modeling. By addressing critical limitations in existing methods and providing a comprehensive pipeline for automated model construction, the proposed framework represents a substantial step toward making CFD-based analyses a practical and reliable tool in clinical practice. Future efforts will focus on addressing current limitations and exploring opportunities to enhance the scalability, robustness, and clinical impact of this approach.

## MATERIALS AND METHODS

### Dataset creation and preprocessing

This study leverages data from two open-source repositories: the “vascular model repository (VMR)”, curated by Wilson *et al.* (*61*), and the “multicenter aortic vessel (AVT)” dataset, released by Radl *et al.* (*62*). The VMR geometries were created by experts using SimVascular through a standardized workflow involving image loading, centerline extraction, cross-sectional contour segmentation, surface reconstruction, branch blending, and mesh export. For the AVT dataset, voxel labels were generated in 3D Slicer through image loading, preprocessing, local thresholding, manual correction (paint/erase), and masked image export, which are already in voxel form. These two datasets have different anatomical focuses: The VMR contains various cardiovascular structures, including the aorta, cerebral arteries, coronary arteries, aortofemoral arteries, and pulmonary arteries. Meanwhile, the AVT dataset comprises only aortic vessels with branches collected from three hospitals: KiTS, RIDER, and Dongyang. For this study, we focused on 32 aortic vessels with supra-aortic branches (e.g., right and left common carotid arteries and right and left subclavian arteries) from the VMR and an additional 16 samples from Dongyang’s hospital in the AVT dataset.

The VMR samples were originally labeled in the form of surface meshes, which necessitated conversion to voxelized volume labels for our segmentation task. To achieve this, we developed a hole-filling algorithm that transforms surface meshes into binary voxel labels. For each voxel in the raw medical image, the algorithm identifies the nearest surface mesh point, computes its normal vector, and calculates the angle between this vector and the distance vector from the voxel to the surface point. Voxels with angles smaller than 90° are classified as “within the vessel,” while those exceeding this threshold are categorized as “outside.” The resulting binary voxel labels maintain the same dimensions as the raw medical images, with one indicating “within the vessel” and zero indicating “outside”, and are stored at the voxel centers.

To enhance segmentation robustness and ensure compatibility with the LoGB-Net model, the raw medical images underwent a series of preprocessing steps. (i) Resampling: The raw images were resampled using cubic interpolation, which increased the resolution by approximately 50%; (ii) clipping and normalization: Image intensity values were clipped between 0 and 500 and then normalized to the range [0, 1]. This clipping range covers 7.2% of the full intensity span and was specifically chosen to suppress extreme outliers (e.g., scanner-related noise and irrelevant backgrounds) while preserving anatomical contrast within the region of interest—the aorta and supra-aortic branches; (iii) background suppression: For training samples, pixel values outside the vessel regions (based on the image labels) were set to zero to focus the model’s attention on vessel features. This step was omitted for testing samples, where image labels are assumed to be unavailable during inference.

To further bolster model generalizability, we applied data augmentation techniques, including random flipping, rotation, and cropping. The cropped dimensions were set to 64 × 64 × 64, substantially smaller than the original image size of 512 × 512 × 128. This approach not only increased the effective number of training samples but also conserved GPU memory during training. During inference, testing images were divided into overlapping cubic subregions of 64 × 64 × 64, which were processed independently by the trained model to predict binary voxel labels. These predictions were subsequently stitched together to reconstruct the full segmentation of the original image. This pipeline ensures that the model can handle high-resolution inputs while maintaining computational efficiency.

### LoGB-Net: A multiscale vascular segmentation model

#### Hierarchical LoG kernels

**Fig. 7.**
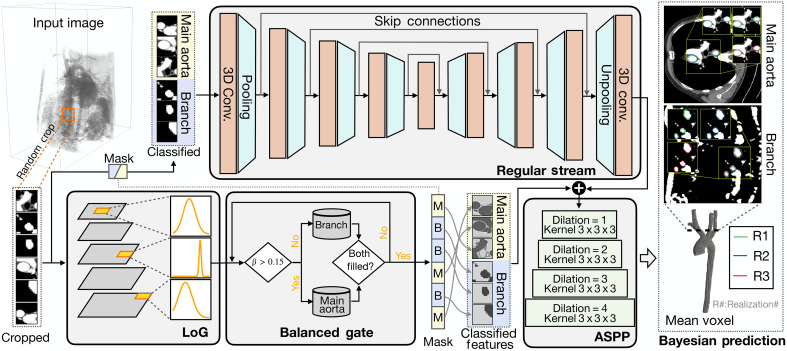
Overview of the LoGB-Net architecture for voxel-based segmentation of aortic vessels. Input medical images are cropped into main aorta and branch regions and processed through the regular stream and LoG stream for feature extraction. A balanced gate ensures equal focus on the main aorta and branch regions. Features are fused via an ASPP module, producing Bayesian predictions with mean segmentation and UQ, capturing anatomically accurate structures.

Our semantic segmentation model, LoGB-Net, features three primary components: a regular stream, a LoG stream, and an atrous spatial pyramid pooling (ASPP) module (*63*), as illustrated in Fig. 7. The pipeline proceeds as follows: The input image is first cropped into small patches, which are passed through the Laplacian-of-Gaussian (LoG) stream to extract feature representations. These feature patches are then classified by the balanced gate into two categories—those containing the main aorta and those containing branch vessels. The classification masks are then reused to categorize the corresponding original image patches, which are subsequently passed into the regular stream. The outputs of the regular stream and the LoG feature patches are then fused and fed into the ASPP module to produce the final voxel-wise segmentations.

The regular stream leverages a 3D CNN U-Net architecture, widely recognized for its efficacy in medical image segmentation tasks. This stream adopts a symmetrical encoder-decoder structure, where the encoder comprises five sequential blocks. Each block contains two 3 × 3 × 3 convolutional layers, a rectified linear unit, and a 2 × 2 × 2 max-pooling layer. To enhance feature fusion and mitigate vanishing gradient issues, skip connections are integrated between corresponding encoder and decoder layers, ensuring that high-resolution spatial features are preserved throughout the network. The LoG stream enhances vessel detection across multiple scales by incorporating hierarchical 3D LoG filters. These filters are mathematically defined as

$$\begin{array}{c} \nabla^{2}G\left(x,y,z\right)=\frac{\partial^{2}G\left(x,y,z\right)}{\partial x^{2}}+\frac{\partial^{2}G\left(x,y,z\right)}{\partial y^{2}}+\frac{\partial^{2}G\left(x,y,z\right)}{\partial z^{2}} \end{array}$$ (1)

where the 3D Gaussian kernel G(x,y,z) is expressed as

$$G\left(x,y,z\right)=\frac{1}{2{\pi \sigma }^{2}}\text{exp}\left[-\frac{x^{2}+y^{2}+z^{2}}{2\sigma^{2}}\right]$$ (2)

Substituting G(x,y,z) into the Laplacian operator results in the LoG filter

$$\nabla^{2}G\left(x,y,z\right)=\frac{x^{2}+y^{2}+z^{2}-2\sigma^{2}}{\sigma^{4}}\text{exp}\left[-\left(x^{2}+y^{2}+z^{2}\right)/2\sigma^{2}\right]$$ (3)

Here, (x,y,z) represents the 3D coordinates of the image voxel, and σ controls the scale of the filter.

The rationale for combining the Laplacian and Gaussian operators lies in their complementary functionalities: The Laplacian operator excels at extracting second-order gradient information from the image, which is crucial for detecting edges and capturing structural details, while the Gaussian prefiltering reduces the impact of noise by smoothing the image. This combination allows the model to retain the essential gradient features for segmentation while minimizing performance degradation caused by the noise sensitivity inherent to the Laplacian operation.

To capture the diverse dimensions of vascular structures in medical images, the LoG stream uses a hierarchical arrangement of filters. Previous studies have demonstrated that the kernel size directly correlates with the size of the target object (*64*). Guided by this insight, we determined optimal kernel sizes of 3, 5, 7, 9, and 11. This five-level configuration [ L(5) ] was empirically validated as optimal. Configurations with fewer LoG layers yielded notably lower segmentation accuracy. We additionally tested a sixth LoG layer using a larger kernel (13 × 13 × 13) and a higher σ (3.0); however, this resulted in performance degradation due to excessive smoothing of fine anatomical details and substantially increased memory consumption by ~86%. These five scales allow the LoGB-Net to effectively detect both large vessels, such as the main aorta, and smaller branches, such as the carotid and subclavian arteries. During training, the kernel sizes and σ values in LoGB-Net can be dynamically further optimized. This dynamic adaptation may enable the model to generalize across varying image resolutions and vessel structures.

#### The Bayesian settings

Medical images often exhibit suboptimal signal quality, with blurred lumen edges and excessive noise. These artifacts make accurately delineating vessel boundaries particularly challenging. To address this issue, we formulate the training process of the LoG module in a Bayesian framework, which can estimate prediction uncertainty due to the presence of noise and data ambiguity, as inspired by similar approaches in Thiagarajan *et al.* (*65*).

In the Bayesian framework, the parameters θ of the LoG module are treated as random variables (RVs). Each parameter is modeled as a Gaussian distribution characterized by a mean β and SD σ. We initialize the prior mean β to zero and set the prior SDs σ to 0.5, 1.0, 1.5, 2.0, and 2.5 for the five respective layers. The posterior distribution given the data $D={\left\{{\left(\text{Image},\text{Label}\right)}_{i}\right\}}_{i=1}^{N}$ is defined by Bayes’ theorem

$$p\left(\theta |D\right)=\frac{p\left(D|\theta \right)p\left(\theta \right)}{\int p\left(D|\theta \right)p\left(\theta \right)d\theta }$$ (4)

where p(θ),p(D∣θ),p(θ∣D) represents the prior, likelihood, and the posterior, respectively. In practice, directly computing the posterior is intractable due to the high dimensionality of the trainable parameter space. Therefore, we approximate the posterior using mean-field variational inference by assuming that the trainable parameters θ are Gaussian RVs. Namely, the prior distributions p(θ) are modeled as standard Gaussian, and the posterior distributions are also Gaussian, parameterized by mean and variances. The variational approximation is optimized by maximizing the evidence lower bound (ELBO)

$$\text{ELBO}=E_{q\left(\theta |D\right)}\left[\text{log}p\left(D|\theta \right)\right]-\text{KL}\left[q\left(\theta |D\right)\|p\left(\theta \right)\right]$$ (5)

where the first term is the expected log-likelihood, measuring how well the model explains the data, and the second term evaluates the Kullback-Leibler divergence, penalizing the deviations of the variational distribution q(θ∣D) from the prior p(θ) . We use the reparameterization trick to sample weights θ, which ensures that the sampling process is differentiable with respect to the ELBO objective, enabling gradient-based optimization.

In addition to the Bayesian ELBO, we incorporate the Dice coefficient loss to optimize segmentation accuracy. The Dice coefficient measures the overlap between the predicted segmentation and the ground truth and is defined as

$$\text{Dice}=\frac{2\sum_{i=1}^{N_{A}}g_{i}o_{i}}{\sum_{i=1}^{N_{A}}g_{i}^{2}+\sum_{i=1}^{N_{A}}o_{i}^{2}}$$ (6)

where $g_{i}$ and $o_{i}$ represent the ground truth and predicted voxel values, respectively. $N_{A}$ denotes the total number of voxels. The Dice loss is expressed as: $L_{\text{Dice}}=1L\text{Dice}$ . The training process involves optimizing a combined loss function that maximizes the ELBO and minimizes the Dice loss

$$\hat{\theta }=\text{arg}\underset{\theta }{\text{min}}\left(L_{\text{Dice}}-\text{ELBO}\right)$$ (7)

To incorporate domain-specific knowledge, we initialize the posterior mean of the Bayesian convolutional layers with precomputed LoG kernels. This initialization biases the model toward detecting vascular structures, facilitating faster convergence and improved performance in early training stages.

#### The balanced gate

During training, medical images are randomly cropped into (64 × 64 × 64) to standardize input dimensions and optimize computational efficiency. However, this approach leads to a severe imbalance in the distribution of training samples, as cubes containing the main aorta vessel are far more prevalent than those containing smaller vascular branches. This imbalance occurs because the main aorta occupies a larger volume in the imaging domain, while smaller branches, such as the supra-aortic vessels, are comparatively limited in size and representation. Without addressing this imbalance, the model tends to prioritize the detection of the main aorta, resulting in suboptimal performance on smaller branches, as observed in preliminary experiments.

To mitigate this issue, a balanced gate was incorporated adjacent to the LoG stream to ensure equitable representation of both large and small vessel regions during training. This mechanism leverages the output of the LoG stream, which is specifically designed to highlight vessel edges. For each input cube, a voxel percentage metric $V_{p}$ is computed, defined as the proportion of voxels in the cube that exceed a threshold value of 0.5

$$V_{p}=\frac{\sum_{i=1}^{N_{c}}1\left(o_{i}>0.5\right)}{N_{c}}$$ (8)

where $v_{i}$ represents the predicted value of the *i*-th voxel in the cube, $N_{c}$ is the total number of voxels in the cube, and 1(⋅) is an indicator function that returns one if the condition is satisfied and zero otherwise. Since the LoG kernels are optimized for edge detection, a higher voxel percentage indicates a higher likelihood of the cube containing larger vessels, such as the main aorta. Conversely, a lower voxel percentage is indicative of smaller branches or regions with fewer vessel features.

The balanced gate classifies cropped image cubes/patches into “main aorta” or “branch” categories in a fully automatic manner. Specifically, given the feature maps produced by the LoG stream, we compute the voxel percentage and compared it against a threshold value of β = 15% to classify the patch as either a main aorta region or a branch vessel. This classification is applied in parallel across multiple patches without user intervention.

Two candidate pools are maintained: one for main aorta patches and one for branch patches. Each pool is iteratively filled until it reaches a target capacity (e.g., 10 patches each). Once full, a balanced training batch is assembled by randomly selecting an equal number of patches (e.g., five per category). After the feature patches are classified, a binary mask, denoted as *M* for “main aorta” and *B* for “branch,” is also used to label corresponding original image patches. The balanced gate mechanism addresses the inherent data imbalance, ensuring that the model is exposed to sufficient training examples of both large and small vascular structures. This approach is particularly critical for achieving high segmentation accuracy across multibranch vascular geometries. The threshold value β = 15% was empirically determined as optimal by evaluating multiple values and selecting the threshold requiring the fewest samples to achieve balanced pool filling, thus maximizing sampling efficiency and class balance.

#### Training, testing, and evaluation metrics

The training and testing of our model, along with other baseline models, were conducted using a combination of two open-source datasets: VMR and AVT. The total dataset was divided into 66% for training and 33% for testing. Specifically, 24 samples were taken from VMR, and 8 samples were obtained from AVT for training purposes. For testing, 16 samples were reserved and evenly distributed between the two data sources. All training experiments were conducted on an NVIDIA GeForce RTX 3080 with 12 gigabytes of memory. Each model was trained for 5000 epochs, requiring ~18 hours. The training process used a learning rate of 1 × 10^−5^and a batch size of 8, optimized via Adam optimizer.

To assess model performance, we used three widely recognized metrics in the image segmentation community: Dice coefficient, ASD, and Hausdorff distance. The Dice coefficient, mathematically expressed in Eq. 6, quantifies the overlap between the predicted segmentation and the corresponding ground truth.

The ASD measures the mean distance between the surface point cloud of predicted segmentation ( $S_{1}$ ) and the ground truth ( $S_{2}$ ), defined as follows

$$\text{ASD}\left(S_{1},S_{2}\right)=\frac{1}{|S_{1}|+|S_{2}|}\left[\sum_{x\in S_{1}}\underset{y\in S_{2}}{\text{min}}\;d\left(x,y\right)+\sum_{y\in S_{2}}\underset{x\in S_{1}}{\text{min}}\;d(y,x)\right]$$ (9)

where $|S_{1}|$ and $|S_{2}|$ represent the number of surface points in the predicted surface and the ground truth segmentations, respectively, and d(x,y) denotes the Euclidean distance between points x and y.

The Hausdorff distance evaluates the maximum surface discrepancy by identifying the largest minimal distance between points on the predicted and ground truth surfaces. It is defined as

$$\begin{array}{c} H\left(S_{1},S_{2}\right)=\text{max}\left\{\underset{x\in S_{1}}{\text{sup}}\underset{y\in S_{2}}{\;\text{inf}\;}d\left(x,y\right),\underset{y\in S_{2}}{\text{sup}}\;\underset{x\in S_{1}}{\text{inf}}\;d(x,y)\right\} \end{array}$$ (10)

where sup and inf denote the supremum and infimum, respectively.

For an optimal segmentation model, the Dice coefficient should approach 1, indicating near-perfect overlap between the predicted and ground truth segmentations. Conversely, smaller values for ASD and Hausdorff distance indicate improved geometric fidelity and minimized surface discrepancies. These metrics collectively provide a robust evaluation framework for comparing the performance of different models on vascular segmentation tasks.

To evaluate the quality of the image at point *a*, we calculate the SNR according to the following formula

$${\text{SNR}}_{a}=\frac{\frac{1}{n}\sum_{i=1}^{n}x_{i}}{\sqrt{\frac{1}{n}\sum_{i=1}^{n}{\left(x_{i}-\frac{1}{n}\sum_{j=1}^{n}x_{j}\right)}^{2}}}$$ (11)

where $x_{i}$ are the voxel values within the 10 × 10 × 10 volume centered at point *a*. This definition is relative SNR in dimensionless form.

### The GNN-LDDMM framework for surface reconstruction and refinement

#### Preprocessing

The preprocessing module begins by reconstructing a triangular surface mesh from the predicted binary voxel image from the LoGB-Net. This is accomplished by extracting the isosurface using the marching cubes algorithm (*66*), a widely used technique for generating 3D surface representations from volumetric data. However, the initial mesh produced by this method often exhibits irregular vertex distributions and a “LEGO-like” blocky appearance due to the voxelized nature of the input. To address these issues, the mesh is resampled using the approximated centroidal voronoi diagram algorithm (*67*), which redistributes vertices to achieve a more uniform and structured layout. This resampling step not only enhances the visual quality of the mesh but also improves its geometric consistency, making it more amenable to subsequent processing stages. To further eliminate the blocky surface, the resampled mesh undergoes a smoothing process implemented with the PyTorch3D package (*68*). This process involves minimizing three regularization terms: normal loss, which aligns neighboring surface normals to reduce abrupt changes in orientation; edge loss, which enforces uniformity in edge lengths to prevent distortions; and Laplacian loss, which smooths the surface by minimizing local curvature variations. Together, these operations produce a refined and well-contoured mesh that preserves anatomical fidelity and is optimized for subsequent deformation processes.

#### GNN-LDDMM surface deformation module

**Fig. 8.**
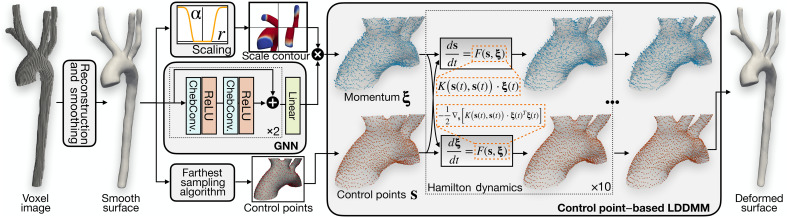
Overview of the GNN-LDDMM module. The GNN-LDDMM module refines the surface mesh using a GNN to predict the initial momentum field, processed through Chebyshev convolution layers. The momentum field drives the LDDMM framework, which iteratively deforms the surface via Hamiltonian dynamics, ensuring smooth and anatomically accurate transformations.

The GNN-LDDMM surface deformation module is designed to refine the alignment of the reconstructed surface mesh with the underlying image background in an unsupervised manner. This module integrates a GNN to predict a vector field on the surface point cloud and a LDDMM algorithm to ensure smooth, anatomically consistent deformations (see Fig. 8). Inspired by (*47*), the LDDMM component is specifically tailored to avoid surface distortions and inaccuracies, which are common in GNN-based deformation approaches.

The GNN component consists of two blocks, each incorporating two Chebyshev convolutional (ChevConv) layers with residual connections. These layers process the point coordinates of the surface mesh and compute a vector field *v* over the surface point cloud. This vector field serves as the initial momentum for the subsequent LDDMM-based deformation.

The LDDMM operates on the initial surface S , which is uniformly subsampled to generate a set of control points ${\left\{s_{i}\right\}}_{i=1}^{N_{s}}$ as detailed in (*69*). Each control point is initialized with a momentum vector ${\left\{\xi_{i}\right\}}_{i=1}^{N_{s}}$ , and together these define a velocity field through kernel interpolation

$$v\left(x\right)=\sum_{i=1}^{N_{s}}K\left(x,s_{i}\right)\cdot \xi_{i}$$ (12)

where *K* is the standard Gaussian kernel

$$K\left(x,y\right)=\text{exp}\left(-\frac{{\left\|x-y\right\|}^{2}}{\sigma^{2}}\right)$$ (13)

The control points and their associated momenta evolve iteratively under Hamiltonian dynamics

$$\left\{\begin{array}{cc} \frac{ds}{\mathrm{dt}} & =K\left[s\left(t\right),s\left(t\right)\right]\cdot \xi \left(t\right),\\ \frac{d\xi }{\mathrm{dt}} & =-\frac{1}{2}\nabla_{s}\left\{K\left[s\left(t\right),s\left(t\right)\right]\cdot \xi {\left(t\right)}^{T}\xi \left(t\right)\right\} \end{array},\;\right.$$ (14)

These equations are numerically integrated using a second-order Runge-Kutta method to compute the time-dependent velocity field v(x,t) . This velocity field governs the deformation of the surface over a discretized time span T=1 , divided into 15 steps. The resulting movement of the surface is described by the following ordinary differential equation (ODE)

$$\frac{dS}{\mathrm{dt}}=v\left(S,t\right)$$ (15)

The deformed surface $\hat{S}$ is obtained by integrating the ODE over the entire time span

$$\hat{S}=\Phi \left(S;s,\xi \right)=\int_{t=0}^{1}v_{s,\xi }\left(S,t\right)\mathrm{dt}$$ (16)

where Φ(S;s,ξ) represents the diffeomorphic transformation, fully parameterized by the initial control points s and momentum ξ.

The integration of the GNN and LDDMM components ensures that the deformation process is both data-driven and anatomically consistent. The GNN generates an initial momentum field that adapts to the specific geometry of the surface, while the LDDMM guarantees that the resulting transformations maintain topological consistency. This combination is particularly effective for handling complex vascular structures, where traditional deformation methods often introduce distortions or fail to align surfaces accurately. A key feature of our approach is a surface deformation algorithm that iteratively aligns the mesh with underlying image gradients. This avoids issues common in manual methods, such as oversmoothing from NURBS fitting or artifacts at branch junctions from Boolean operations. By optimizing every surface vertex based on the full 3D image, our method enhances geometric fidelity and preserves fine anatomical details. A key feature of our approach is a surface deformation algorithm that iteratively aligns the mesh with underlying image gradients. This avoids issues common in manual methods, such as oversmoothing from NURBS fitting or artifacts at branch junctions from Boolean operations. By optimizing every surface vertex based on the full 3D image, our method enhances geometric fidelity and preserves fine anatomical details. Unlike tools such as SimVascular, which depend on manual annotations and are susceptible to operator variability, our pipeline produces anatomically accurate, simulation-ready models with minimal user intervention.

#### The scaling gate

One of the major challenges during the deformation process is the severe distortion observed at the inlets and outlets of the aorta. These regions are not the primary focus of the alignment process, which primarily targets the vessel wall, leading to control points near the inlets and outlets being optimized to arbitrary locations. Such behavior often results in undesirable deformations at these boundary regions. To mitigate this issue, we implemented a scaling gate to restrict the movement of surface points, specifically at the inlets and outlets.

Before initiating the deformation, a scalar field ${\left\{\alpha \left(x_{i}\right)\right\}}_{i=1}^{N_{s}}$ is computed over the control point cloud. This scalar field determines the degree of mobility for each surface point based on its location relative to the inlets and outlets. Specifically, for points located within the inlets or outlets, the geodesic distance from the center point to the boundary (geodesic radius) is calculated. Then the scalar field α(x) is defined as

$$\begin{array}{c} \begin{array}{c} \alpha \left(x\right)=\\ \left\{\begin{array}{cc} 0, & |x-x_{c}^{i}|<r^{i}\;\forall i\in \left[0,N_{I/O}\right],\\ 1-\text{exp}\left[-\frac{{\left(|x-x_{c}^{i}|-r^{i}\right)}^{2}}{2\sigma^{2}}\right], & r^{i}\leq |x-x_{c}^{i}|<r^{i}+3E\;\forall i\in \left[0,N_{I/O}\right],\\ 1, & |x-x_{c}^{i}|\geq r^{i}+3\sigma \;\forall i\in \left[0,N_{I/O}\right] \end{array}\right. \end{array} \end{array}$$ (17)

where ${\left\{x_{c}^{i}\right\}}_{i=1}^{N_{I/O}}$ is the center point of the inlets and outlets, ${\left\{r^{i}\right\}}_{i=1}^{N_{I/O}}$ is the geodesic radius of the inlets and outlets, $N_{I/O}$ is the total number of inlets and outlets in the vascular tree, and σ controls the width of the buffer zone, ensuring a smooth transition between fully immobilized and fully mobile regions. Namely, for points on the inlets or outlets, α = 0, effectively immobilizing these points; for points along the vessel wall, α = 1, allowing full mobility; for points in the transitional region, α smoothly transitions from 1 to 0 using a truncated Gaussian function to ensure a gradual gradation in mobility constraints.

#### Unsupervised training based on input medical imaging

The training process for the surface deformation module is designed to operate in an unsupervised manner, relying solely on the input medical imaging data to drive the alignment and ensure high-quality mesh deformation. The objective function consists of two main components: an energy term that quantifies the alignment between the deformed surface and the input medical image and surface regularization terms that ensure the geometric quality of the resulting mesh. The total loss function is formulated as

$$\begin{array}{c} L_{\text{total}}=-w_{1}\text{log}\\ \left[\sum_{i}^{N_{A}}G\left(s_{i}\right)\right]+w_{2}L_{\text{normal}}+w_{3}L_{\text{edge}}+w_{4}L_{\text{laplacian}} \end{array}$$ (18)

where $G\left(s_{i}\right)$ represents the image gradient magnitude at the *i*-th surface point $s_{i}$ and $N_{A}$ is the total number of points on the surface mesh. The terms $L_{\text{normal}}$ , $L_{\text{edge}}$ , and $L_{\text{laplacian}}$ are the surface regularization terms, and $w_{1},w_{2},w_{3},w_{4}$ are the associated weights.

The first term, referred to as the misalignment energy, is defined as the negative logarithm of the sum of image gradient magnitudes over the surface point cloud. Minimizing this term encourages the surface points to move toward regions in the medical image with the largest gradients, corresponding to anatomical boundaries or regions of high contrast. This ensures that the deformed surface aligns accurately with the image data, capturing the underlying anatomical structure.

The second term, $L_{\text{normal}}$ , referred to as the normal loss, penalizes deviations in the orientation of adjacent surface normals. By encouraging parallelism between neighboring normals, this term prevents abrupt changes in surface orientation and mitigates the risk of folding or kinks in the mesh

$$L_{\text{normal}}=\frac{1}{N_{\text{IE}}}\sum_{i=1}^{N_{\text{IE}}}\left(1-n_{1}\cdot n_{2}\right)$$ (19)

where $N_{\text{IE}}$ is the total number of internal edges on a triangulated mesh and $n_{1}$ and $n_{2}$ are the surface normals of the two neighboring triangular elements $e_{i}$ and $e_{j}$.

The third term, $L_{\text{edge}}$ , is the edge loss, which ensures uniformity in edge lengths across the mesh. This term is defined as

$$L_{\text{edge}}=\frac{1}{N_{E}}\sum_{e\in E}{\left(∥p_{e}∥-\bar{l}\right)}^{2}$$ (20)

where E is the set of all edges in the mesh, $p_{e}$ represents the edge vector, $\bar{l}$ is the average edge length, and $N_{E}$ is the total number of edges. Minimizing this term reduces regional distortions and improves the uniformity of the mesh geometry.

The last term, the Laplacian loss $L_{\text{laplacian}}$ (*70*), enhances the smoothness of the surface by penalizing deviations from local surface curvature. It is expressed as

$$\begin{array}{c} L_{\text{laplacian}}=\frac{1}{N_{A}}\sum_{i=1}^{N_{A}}{∥s_{i}-\frac{1}{|N\left(i\right)|}\sum_{j\in N\left(i\right)}s_{j}∥}^{2} \end{array}$$ (21)

where $s_{i}$ is the position of the *i*-th surface point and N(i) is its set of neighbors. This term smooths the surface by ensuring that each point’s position aligns closely with the average position of its neighbors, thereby reducing sharp irregularities.

For all samples, the weights of the loss terms are set to $w_{1}=1.0$ , $w_{2}=0.2$ , $w_{3}=0.01$ , and $w_{4}=0.1$ . These values are chosen to balance alignment with the image background and the geometric quality of the surface mesh. By minimizing $L_{\text{total}}$ , the optimization process achieves accurate surface alignment while maintaining a high-quality mesh that is ready for downstream CFD/FSI simulations. The generated surfaces are further postprocessed into volumetric meshes compatible with CFD simulations (see details in the Supplementary Materials).

In summary, the proposed method realizes a fully automated image-to-voxel-to-geometry pipeline. It segments raw medical images using LoGB-Net and then refines the output surfaces with a GNN-LDDMM–based deformation guided by image gradients, producing CFD-ready meshes. The Bayesian formulation enables UQ, enhancing robustness and supporting informed clinical decision-making. In addition, the unsupervised surface refinement reduces manual artifacts, further improving accuracy and reproducibility.

## Supplementary Materials

### This PDF file includes:

Supplementary Materials and Methods Supplementary Text Figs. S1 to S4 Table S1 References
