# Supplementary material for: AI-powered automated model construction for patient-specific CFD simulations of aortic flows
Source: Sci Adv. 2025 Sep 5;11(36):eadw2825. doi: 10.1126/sciadv.adw2825 (PMC12412661; doi:10.1126/sciadv.adw2825)
Supplement: Supplementary file 1 — Supplementary Materials and Methods Supplementary Text Figs. S1 to S4 Table S1 References [file sciadv.adw2825_sm.pdf]

Science Advances
AAAS

Supplementary Materials for

AI-powered automated model construction for patient-specific CFD simulations of aortic flows

Pan Du *et al.*

Corresponding author: Jian-Xun Wang, jw2837@cornell.edu

Sci. Adv. **11**, eadw2825 (2025)
DOI: 10.1126/sciadv.adw2825

This PDF file includes:

Supplementary Materials and Methods
Supplementary Text
Figs. S1 to S4
Table S1
References

Materials and Methods

Details of data preprocessing, model architectures, and training/testing protocols are fully described in the Materials and Methods section of the main text. Here, we provide additional information on the post-processing of the deformed surface meshes and the downstream CFD simulation settings.

Post-processing

The post-processing pipeline transforms the deformed surfaces produced by the deformation module into volumetric meshes suitable CFD simulations. Figure S2 illustrates the step-by-step procedure: starting from a watertight surface mesh, the position and orientation of clipping planes are automatically determined based on the inlet/outlet points and their associated normal vectors. These planes are then applied to trim the mesh tips, ensuring open-ended aortic surface. The modified mesh is subsequently capped, volumetrically meshed, and exported for CFD simulations and analysis.

CFD simulation settings

We conduct CFD simulations on the post-processed mesh by solving the steady-state, incompressible Navier–Stokes equations:

$$\begin{cases} \nabla \cdot \mathbf{u} = 0, \\ (\mathbf{u} \cdot \nabla)\mathbf{u} = -\nabla p + \nu \nabla^2 \mathbf{u}, \end{cases} \tag{S1}$$

where $\mathbf{u}$ denotes the velocity field, p is the pressure, and ν is the kinematic viscosity.

These equations were discretized and solved using the simpleFoam solver in OpenFOAM, employing the SIMPLE algorithm (*74*). Simulations were executed in parallel across multiple CPU cores using the OpenMPI framework (*75*). A parabolic inlet velocity profile with a peak velocity of 1 $\mathrm{m\,s^{-1}}$ was prescribed, along with a zero-gradient pressure condition. At the outlet, a fixed pressure and an outflow velocity condition were applied to allow for natural flow development. Vessel walls were treated as rigid and stationary, with no-slip boundary conditions imposed. Upon convergence, key hemodynamic quantities—including velocity magnitude, pressure distribution, and WSS—were extracted for analysis.

Supplementary Text

Illustration of manual segmentation artifacts

To illustrate operator-dependent artifacts and inter-observer variability in manual segmentation, we asked a third-party segmentation expert (operator 2) to manually segment an aorta sample from the VM dataset and compared it to the label from the original repository (operator 1). We visualized this inter-observer error in figure S1. The results show clear discrepancies in both surface and cross-sectional views. Specifically, in the middle panel, which shows a cross-section near the junction between the main aorta and its branches, the image gradient highlights a bright ring corresponding to the vessel wall. Operator 1's segmentation (cyan) lies slightly inside this bright ring, whereas Operator 2's segmentation (red) lies outside of it. This misalignment reflects differing interpretations of the vessel boundary between the two operators. In the right panel, which shows a cross-section at the LCCA, Operator 2's segmentation (red) deviates notably from the bright ring seen in the image background, indicating poor alignment with the anatomical boundary. Additionally, the shapes of the segmentations from Operator 1 and Operator 2 differ substantially despite both being derived from the same CT image. These observations highlight the inherent subjectivity and variability in manual segmentation.

Effect of geometry variation on CFD results

The geometry of the surface mesh plays a critical role in determining the outcome of CFD simulations. To demonstrate this, we post-processed the output of the "no scaling" and "no LDDMM" cases in Figure 6 and ran CFD simulations. Figure S3 compares the CFD results obtained from these modified geometries.

Geometrically, the "no scaling" and "no LDDMM" meshes display markedly shortened inlet and outlet sections, particularly noticeable in the supra-aortic branches. These differences in geometry produce notable variations in simulated pressure and wall WSS fields. In both cases, upstream pressures are substantially higher in the ascending aorta and arch regions compared to the full model. WSS differences, while generally smaller, are concentrated near vessel termini. This is expected, as WSS is primarily influenced by local flow patterns, which are more dependent on local geometry than on branch length. The pressure differences are even more pronounced in the

“no LDDMM” case due to the more severely truncated branches. These results clearly demonstrate the critical influence of geometric fidelity on CFD predictions and highlight the importance of including both scaling and LDDMM deformation steps in our pipeline.

Comparison between LoGB-Net and SeqSeg

To rigorously compare our LoGB-Net against SeqSeg, we conducted comparative segmentation experiments using the same training and testing sets. The results of one test sample from AVT dataset are presented in figure S4. The leftmost column shows the entire true vascular geometry, covering both the aortic arch and abdominal regions. The segmentation from LoGB-Net (second column) accurately captures most of the aortic structure but misses a few thinner branches, such as the upper bifurcations of the left/right common carotid arteries and distal iliac arteries, since these regions are not considered in our training labels.

For SeqSeg, we explored multiple parameter configurations, highlighting two key inputs: the seed point (blue), marking the start of segmentation, and the direction point (orange), guiding initial tracking. Four representative trials are presented (rightmost columns). Notably, SeqSeg demonstrated the ability to identify vessel segments extending beyond those labeled in the training data, illustrating its excellent generalization capabilities. However, SeqSeg’s performance was found to be highly sensitive to the initial manual parameters. Trial 4 achieved the best segmentation, capturing most of the aorta effectively but still missing some upper-branch details. Quantitative evaluation using Dice scores is summarized in Table S1. LoGB-Net achieved a Dice score of 0.9264, closely matched by the best SeqSeg trial (0.9248). The other SeqSeg trials yielded Dice scores of 0.8530, 0.3430, and 0.1422 (trials 3, 2, and 1, respectively).

In summary, both LoGB-Net and SeqSeg (in optimal configuration) delivered comparable segmentation performance. However, each approach offers distinct advantages and disadvantages:

- SeqSeg excels at generalizing beyond annotated vessel segments but requires user-defined seed points, direction inputs, and careful hyperparameter tuning, making it inherently semi-automatic. Additionally, the training data used in SeqSeg relies on manually created centerlines and selective patch extraction, which is still labor-intensive.

- In contrast, LoGB-Net is fully automatic, requires no manual inputs or hyperparameter tuning,

and uniquely provides UQ—features critical for clinical usability and reproducibility. Like all data-driven methods, LoGB-Net's generalizability is inherently constrained by training data diversity and quality.

We view these two methodologies as complementary rather than competing. A particularly promising future direction is the integration of LoGB-Net's robust voxel-based segmentation and UQ with SeqSeg's vessel-tracking algorithm to create a hybrid approach combining accuracy, automation, and generalization.

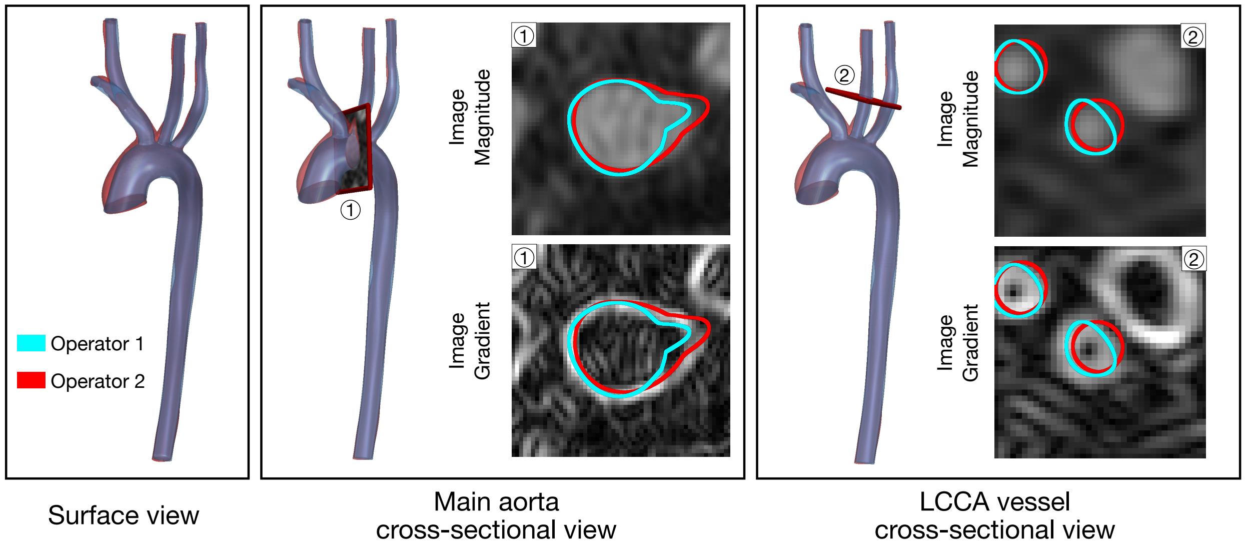

Figure S1: **Comparison of manual segmentation output from two independent operators using SimVascular.**

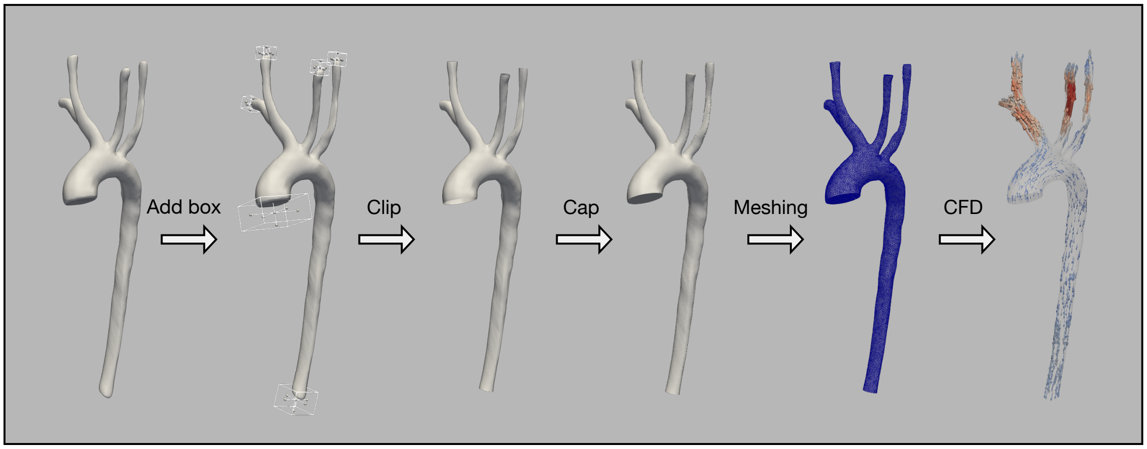

Figure S2: **Post-processing pipeline: from deformed mesh to CFD simulation.**

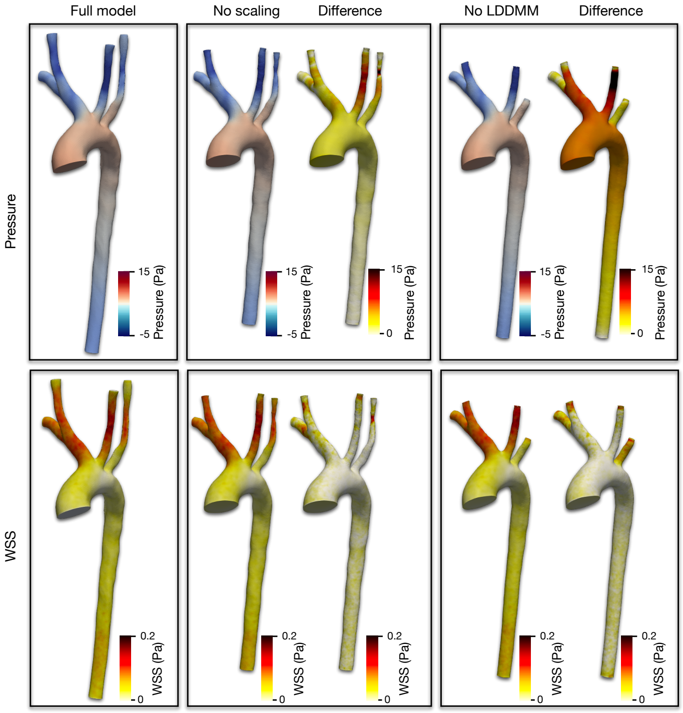

Figure S3: **Comparison of CFD results for meshes with and without scaling.**

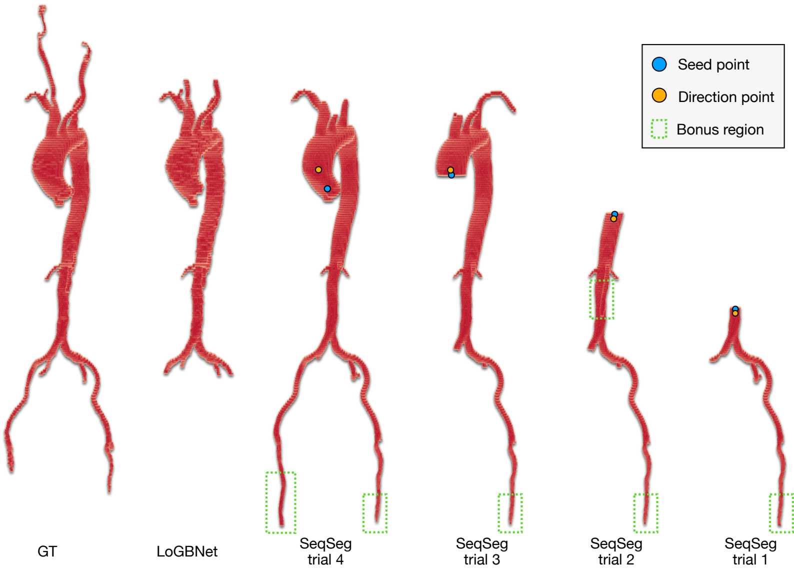

Figure S4: **Comparison of LoGB-Net and SeqSeg segmentation performance on an unseen AVT sample.**

Table S1: **Quantitative comparison between LoGB-Net and SeqSeg segmentations.**

	LoGB-Net	SeqSeg(trail4)	SeqSeg(trail3)	SeqSeg(trail2)	SeqSeg(trail1)
Dice	0.9264	0.9248	0.8530	0.3430	0.1422

REFERENCES AND NOTES

1. B. Şahin, G. İlgün, Risk factors of deaths related to cardiovascular diseases in World Health Organization (WHO) member countries. *Health Soc. Care Community* **30**, 73–80 (2022).

2. D. A. Steinman, Image-based computational fluid dynamics modeling in realistic arterial geometries. *Ann. Biomed. Eng.* **30**, 483–497 (2002).

3. C. A. Taylor, C. Figueroa, Patient-specific modeling of cardiovascular mechanics. *Annu. Rev. Biomed. Eng.* **11**, 109–134 (2009).

4. R. A. Gray, P. Pathmanathan, Patient-specific cardiovascular computational modeling: Diversity of personalization and challenges. *J. Cardiovasc. Transl. Res.* **11**, 80–88 (2018).

5. A. Updegrove, N. M. Wilson, J. Merkow, H. Lan, A. L. Marsden, S. C. Shadden, SimVascular: An open source pipeline for cardiovascular simulation. *Ann. Biomed. Eng.* **45**, 525–541 (2017).

6. P. Du, X. Zhu, J.-X. Wang, Deep learning-based surrogate model for three-dimensional patient-specific computational fluid dynamics. *Phys. Fluids* **34**, 081906 (2022).

7. A. Arzani, J.-X. Wang, M. S. Sacks, S. C. Shadden, Machine learning for cardiovascular biomechanics modeling: Challenges and beyond. *Ann. Biomed. Eng.* **50**, 615–627 (2022).

8. K. Valen-Sendstad, A. W. Bergersen, Y. Shimogonya, L. Goubergrits, J. Bruening, J. Pallares, S. Cito, S. Piskin, K. Pekkan, A. J. Geers, I. Larrabide, S. Rapaka, V. Mihalef, W. Fu, A. Qiao, K. Jain, S. Roller, K.-A. Mardal, R. Kamakoti, T. Spirka, N. Ashton, A. Revell, N. Aristokleous, J. G. Houston, M. Tsuji, F. Ishida, P. G. Menon, L. D. Browne, S. Broderick, M. Shojima, S. Koizumi, M. Barbour, A. Aliseda, H. G. Morales, T. Lefèvre, S. Hodis, Y. M. Al-Smadi, J. S. Tran, A. L. Marsden, S. Vaippummadhom, G. A. Einstein, A. G. Brown, K. Debus, K. Niizuma, S. Rashad, S.-I. Sugiyama, M. O. Khan, A. R. Updegrove, S. C. Shadden, B. M. W. Cornelissen, C. B. L. M. Majoie, P. Berg, S. Saalfield, K. Kono, D. A. Steinman, Real-world variability in the prediction of intracranial aneurysm wall shear stress: The 2015 international aneurysm CFD challenge. *Cardiovasc. Eng. Technol.* **9**, 544–564 (2018).

9. P. Du, J.-X. Wang, Reducing geometric uncertainty in computational hemodynamics by deep learning-assisted parallel-chain MCMC. *J. Biomech. Eng.* **144**, 121009 (2022).

10. A. Ajam, A. A. Aziz, V. S. Asirvadam, A. S. Muda, I. Faye, S. J. S. Gardezi, A review on segmentation and modeling of cerebral vasculature for surgical planning. *IEEE Access* **5**, 15222–15240 (2017).

11. D. Rueckert, P. Burger, S. Forbat, R. Mohiaddin, G.-Z. Yang, Automatic tracking of the aorta in cardiovascular MR images using deformable models. *IEEE Trans. Med. Imaging* **16**, 581–590 (1997).

12. B. Das, Y. Mallya, S. Srikanth, R. Malladi, "Aortic thrombus segmentation using narrow band active contour model," in *2006 International Conference of the IEEE Engineering in Medicine and Biology Society* (IEEE, 2006), pp. 408–411.

13. K. Krissian, J. M. Carreira, J. Esclarin, M. Maynar, Semi-automatic segmentation and detection of aorta dissection wall in MDCT angiography. *Med. Image Anal.* **18**, 83–102 (2014).

14. Y. Wang, F. Seguro, E. Kao, Y. Zhang, F. Faraji, C. Zhu, H. Haraldsson, M. Hope, D. Saloner, J. Liu, Segmentation of lumen and outer wall of abdominal aortic aneurysms from 3D black-blood MRI with a registration based geodesic active contour model. *Med. Image Anal.* **40**, 1–10 (2017).

15. H. Ling, J. Gao, A. Kar, W. Chen, S. Fidler, "Fast interactive object annotation with curve-gcn," in *Proceedings of the IEEE/CVF conference on computer vision and pattern recognition* (IEEE, 2019), pp. 5257–5266.

16. M. E. Leventon, W. E. L. Grimson, O. Faugeras, "Statistical shape influence in geodesic active contours," in *5th IEEE EMBS International Summer School on Biomedical Imaging* (IEEE, 2002), pp. 8.

17. L. He, Z. Peng, B. Everding, X. Wang, C. Y. Han, K. L. Weiss, W. G. Wee, A comparative study of deformable contour methods on medical image segmentation. *Image Vis. Comput.* **26**, 141–163 (2008).

18. A. Jorstad, B. Nigro, C. Cali, M. Wawrzyniak, P. Fua, G. Knott, NeuroMorph: A toolset for the morphometric analysis and visualization of 3D models derived from electron microscopy image stacks. *Neuroinformatics* **13**, 83–92 (2015).

19. T. McInerney, D. Terzopoulos, A dynamic finite element surface model for segmentation and tracking in multidimensional medical images with application to cardiac 4D image analysis. *Comput. Med. Imaging Graph.* **19**, 69–83 (1995).

20. D. Terzopoulos, A. Witkin, M. Kass, Constraints on deformable models: Recovering 3D shape and nonrigid motion. *Artif Intell* **36**, 91–123 (1988).

21. D. Terzopoulos, A. Witkin, M. Kass, Symmetry-seeking models and 3D object reconstruction. *Int. J. Comput. Vis.* **1**, 211–221 (1988).

22. F. Lareyre, C. Adam, M. Carrier, C. Dommerc, C. Mialhe, J. Raffort, A fully automated pipeline for mining abdominal aortic aneurysm using image segmentation. *Sci. Rep.* **9**, 13750 (2019).

23. S. Bidhult, E. Hedström, M. Carlsson, J. Töger, K. Steding-Ehrenborg, H. Arheden, A. H. Aletras, E. Heiberg, A new vessel segmentation algorithm for robust blood flow quantification from two-dimensional phase-contrast magnetic resonance images. *Clin. Physiol. Funct. Imaging* **39**, 327–338 (2019).

24. L. Antiga, M. Piccinelli, L. Botti, B. Ene-Iordache, A. Remuzzi, D. A. Steinman, An image-based modeling framework for patient-specific computational hemodynamics. *Med. Biol. Eng. Comput.* **46**, 1097–1112 (2008).

25. S. Osher, R. Fedkiw, K. Piechor, Level set methods and dynamic implicit surfaces. *Appl. Mech. Rev.* **57**, B15–B15 (2004).

26. P. Volonghi, D. Tresoldi, M. Cadioli, A. M. Usuelli, R. Ponzini, U. Morbiducci, A. Esposito, G. Rizzo, Automatic extraction of three-dimensional thoracic aorta geometric model from phase contrast MRI for morphometric and hemodynamic characterization. *Magn. Reson. Med.* **75**, 873–882 (2016).

27. S. Kurugol, C. E. Come, A. A. Diaz, J. C. Ross, G. L. Kinney, J. L. Black-Shinn, J. E. Hokanson, M. J. Budoff, G. R. Washko, R. San Jose Estepar, Automated quantitative 3D analysis of aorta size, morphology, and mural calcification distributions. *Med. Phys.* **42**, 5467–5478 (2015).

28. F. Zhuge, G. D. Rubin, S. Sun, S. Napel, An abdominal aortic aneurysm segmentation method: Level set with region and statistical information. *Med. Phys.* **33**, 1440–1453 (2006).

29. C. Chen, C. Qin, H. Qiu, G. Tarroni, J. Duan, W. Bai, D. Rueckert, Deep learning for cardiac image segmentation: A review. *Front. Cardiovasc. Med.* **7**, 25 (2020).

30. D. Jia, X. Zhuang, Learning-based algorithms for vessel tracking: A review. *Comput. Med. Imaging Graph.* **89**, 101840 (2021).

31. J. M. Wolterink, T. Leiner, M. A. Viergever, I. Išgum, "Dilated convolutional neural networks for cardiovascular MR segmentation in congenital heart disease," in *International Workshop on Reconstruction and Analysis of Moving Body Organs* (Springer, 2016), pp. 95–102.

32. W. Bai, H. Suzuki, C. Qin, G. Tarroni, O. Oktay, P. M. Matthews, D. Rueckert, "Recurrent neural networks for aortic image sequence segmentation with sparse annotations," in *Proceedings of the Medical Image Computing and Computer Assisted Intervention–MICCAI 2018: 21st International Conference, Part IV 11* (Springer, 2018), pp. 586–594.

33. Q. Xia, Y. Yao, Z. Hu, A. Hao, "Automatic 3D atrial segmentation from GE-MRIs using volumetric fully convolutional networks," in *Statistical Atlases and Computational Models of the Heart. Atrial Segmentation and LV Quantification Challenges: 9th International Workshop, STACOM 2018* (Springer, 2019), pp. 211–220.

34. D. M. Vigneault, W. Xie, C. Y. Ho, D. A. Bluemke, J. A. Noble, Ω-net (Omega-Net): Fully automatic, multi-view cardiac MR detection, orientation, and segmentation with deep neural networks. *Med. Image Anal.* **48**, 95–106 (2018).

35. J. M. Wolterink, T. Leiner, M. A. Viergever, I. Išgum, "Automatic segmentation and disease classification using cardiac cine MR images," in *Statistical Atlases and Computational*

Models of the Heart. ACDC and MMWHS Challenges: 8th International Workshop, STACOM 2017 (Springer, 2018), pp. 101–110.

36. C. F. Baumgartner, L. M. Koch, M. Pollefeys, E. Konukoglu, "An exploration of 2D and 3D deep learning techniques for cardiac MR image segmentation," in *Statistical Atlases and Computational Models of the Heart. ACDC and MMWHS Challenges: 8th International Workshop, STACOM 2017* (Springer, 2018), pp. 111–119.

37. T. S. Newman, H. Yi, A survey of the marching cubes algorithm. *Comput. Graph.* **30**, 854–879 (2006).

38. J. Zhao, J. Zhao, S. Pang, Q. Feng, Segmentation of the true lumen of aorta dissection via morphology-constrained stepwise deep mesh regression. *IEEE Trans. Med. Imaging* **41**, 1826–1836 (2022).

39. U. Wickramasinghe, P. Fua, G. Knott, "Deep active surface models," in *Proceedings of the IEEE/CVF Conference on Computer Vision and Pattern Recognition* (IEEE, 2021), pp. 11652–11661.

40. F. Kong, N. Wilson, S. Shadden, A deep-learning approach for direct whole-heart mesh reconstruction. *Med. Image Anal.* **74**, 102222 (2021).

41. F. Bongratz, A.-M. Rickmann, S. Pölsterl, C. Wachinger, "Vox2cortex: Fast explicit reconstruction of cortical surfaces from 3D MRI scans with geometric deep neural networks," in *Proceedings of the IEEE/CVF Conference on Computer Vision and Pattern Recognition* (IEEE, 2022), pp. 20773–20783.

42. U. Wickramasinghe, E. Remelli, G. Knott, P. Fua, "Voxel2mesh: 3D mesh model generation from volumetric data," in *Proceedings of the Medical Image Computing and Computer Assisted Intervention–MICCAI 2020: 23rd International Conference, Part IV 23* (Springer, 2020), pp. 299–308.

43. B. Deng, Y. Yao, R. M. Dyke, J. Zhang, A survey of non-rigid 3D registration. *Comput. Graph. Forum* **41**, 559–589 (2022).

44. M. Eisenberger, D. Novotny, G. Kerchenbaum, P. Labatut, N. Neverova, D. Cremers, A. Vedaldi, "Neuromorph: Unsupervised shape interpolation and correspondence in one go," in *Proceedings of the IEEE/CVF Conference on Computer Vision and Pattern Recognition* (IEEE, 2021), pp. 7473–7483.

45. B. B. Amor, S. Arguillère, L. Shao, ResNet-LDDMM: Advancing the LDDMM framework using deep residual networks. *IEEE Trans. Pattern Anal. Mach. Intell.* **45**, 3707–3720 (2022).

46. M. Eisenberger, Z. Lahner, D. Cremers, "Smooth shells: Multi-scale shape registration with functional maps," in *Proceedings of the IEEE/CVF Conference on Computer Vision and Pattern Recognition* (IEEE, 2020), pp. 12265–12274.

47. M. F. Beg, M. I. Miller, A. Trouvé, L. Younes, Computing large deformation metric mappings via geodesic flows of diffeomorphisms. *Int. J. Comput. Vis.* **61**, 139–157 (2005).

48. A. Updegrove, N. M. Wilson, S. C. Shadden, Boolean and smoothing of discrete polygonal surfaces. *Adv. Eng. Softw.* **95**, 16–27 (2016).

49. D. An, P. Du, P. Gu, J.-X. Wang, C. Wang, "Hierarchical LoG Bayesian neural network for enhanced aorta segmentation," in *Proceedings of IEEE International Symposium on Biomedical Imaging* (IEEE, 2025), pp. 1–5.

50. T.-Y. Lin, P. Dollar, R. Girshick, K. He, B. Hariharan, S. J. Belongie, "Feature pyramid networks for object detection," in *Proceedings of IEEE Conference on Computer Vision and Pattern Recognition* (IEEE, 2017), pp. 936–944.

51..Ç içek, A. Abdulkadir, S. S. Lienkamp, T. Brox, O. Ronneberger, "3D U-Net: Learning dense volumetric segmentation from sparse annotation," in *Proceedings of International Conference on Medical Image Computing and Computer Assisted Interventions* (Springer, 2016), pp. 424–432.

52. H. Zhao, J. Shi, X. Qi, X. Wang, J. Jia, "Pyramid scene parsing network," in *Proceedings of IEEE Conference on Computer Vision and Pattern Recognition* (IEEE, 2017), pp. 2881–2890.

53. F. Isensee, P. F. Jaeger, S. A. A. Kohl, J. Petersen, K. H. Maier-Hein, nnU-Net: A self-configuring method for deep learning-based biomedical image segmentation. *Nat. Methods* **18**, 203–211 (2021).

54. O. Oktay, J. Schlemper, L. L. Folgoc, M. Lee, M. Heinrich, K. Misawa, K. Mori, S. McDonagh, N. Y. Hammerla, B. Kainz, B. Glocker, D. Rueckert, Attention U-Net: Learning where to look for the pancreas. arXiv:1804.03999 [cs.CV] (2018).

55. X. Huang, Z. Deng, D. Li, X. Yuan, MISSFormer: An effective medical image segmentation transformer. arXiv:2109.07162 (2021).

56. H. Cao, Y. Wang, J. Chen, D. Jiang, X. Zhang, Q. Tian, M. Wang, "Swin-Unet: Unet-like pure transformer for medical image segmentation," in *Proceedings of European Conference on Computer Vision Workshops* (Springer, 2023), pp. 205–218.

57. J. Chen, Y. Lu, Q. Yu, X. Luo, E. Adeli, Y. Wang, L. Lu, A. L. Yuille, Y. Zhou, TransUNet: Transformers make strong encoders for medical image segmentation. arXiv:2102.04306 [cs.CV] (2021).

58. A. Hatamizadeh, Y. Tang, V. Nath, D. Yang, A. Myronenko, B. Landman, H. R. Roth, D. Xu, "UNETR: Transformers for 3D medical image segmentation," in *Proceedings of IEEE Winter Conference on Applications of Computer Vision* (IEEE, 2022), pp. 574–584.

59. A. Shaker, M. Maaz, H. Rasheed, S. Khan, M.-H. Yang, F. S. Khan, UNETR++: Delving into efficient and accurate 3D medical image segmentation (IEEE, 2023), pp. 3377–3390.

60. N. Sveinsson Cepero, S. C. Shadden, SeqSeg: Learning local segments for automatic vascular model construction. *Ann. Biomed. Eng.* **53**, 158–179 (2025).

61. N. M. Wilson, A. K. Ortiz, A. B. Johnson, The vascular model repository: A public resource of medical imaging data and blood flow simulation results. *J. Med. Devices* **7**, 0409231 (2013).

62. L. Radl, Y. Jin, A. Pepe, J. Li, C. Gsaxner, F.-H. Zhao, J. Egger, AVT: Multicenter aortic vessel tree CTA dataset collection with ground truth segmentation masks. *Data Brief* **40**, 107801 (2022).

63. L.-C. Chen, G. Papandreou, I. Kokkinos, K. Murphy, A. L. Yuille, DeepLab: Semantic image segmentation with deep convolutional nets, atrous convolution, and fully connected CRFs. *IEEE Trans. Pattern Anal. Mach. Intell.* **40**, 834–848 (2018).

64. K. A. Vermeer, F. M. Vos, H. G. Lemij, A. M. Vossepoel, A model based method for retinal blood vessel detection. *Comput. Biol. Med.* **34**, 209–219 (2004).

65. P. Thiagarajan, P. Khairnar, S. Ghosh, Explanation and use of uncertainty quantified by Bayesian neural network classifiers for breast histopathology images. *IEEE Trans. Med. Imaging* **41**, 815–825 (2022).

66. W. E. Lorensen, H. E. Cline, Marching cubes: A high resolution 3D surface construction algorithm, in *Seminal Graphics: Pioneering Efforts that Shaped the Field* (ACM, 1998), pp. 347–353.

67. S. Valette, J.-M. Chassery, Approximated centroidal voronoi diagrams for uniform polygonal mesh coarsening. *Comput. Graph. Forum* **23**, 381–389 (2004).

68. N. Ravi, J. Reizenstein, D. Novotny, T. Gordon, W.-Y. Lo, J. Johnson, G. Gkioxari, Accelerating 3D deep learning with PyTorch3D. arXiv:2007.08501 [cs.CV] (2020).

69. C. R. Qi, L. Yi, H. Su, L. J. Guibas, Pointnet++: Deep hierarchical feature learning on point sets in a metric space. *Adv. Neural Inf. Process. Syst.* **30**, 5105–5114 (2017).

70. A. Nealen, T. Igarashi, O. Sorkine, M. Alexa, "Laplacian mesh optimization," in *Proceedings of the 4th International Conference on Computer Graphics and Interactive Techniques in Australasia and Southeast Asia* (ACM, 2006), pp. 381–389.

71. P. Du, D. An, C. Wang, J.-X. Wang, Dataset for AI-powered automated model construction for patient-specific CFD simulations of aortic flows (version 1) [Dataset]. (Zenodo, 2025); https://doi.org/10.5281/zenodo.16663170.

72. P. Du, D. An, C. Wang, J.-X. Wang, Automatic segmentation with deformation model (Version 1.0) [Computer software] Zenodo, (2025). https://doi.org/10.5281/zenodo.16923770.

73. D. An, P. Du, J.-X. Wang, C. Wang, Hierarchical LoG Bayesian neural network for enhanced aorta segmentation (version 1.0) [Computer software] Zenodo, (2025). https://doi.org/10.5281/zenodo.16927932.

74. S. V. Patankar, D. B. Spalding, "A calculation procedure for heat, mass and momentum transfer in three-dimensional parabolic flows," in *Numerical Prediction of Flow, Heat Transfer, Turbulence and c#ombustion* (Elsevier, 1983), pp. 54–73.

75. R. L. Graham, T. S. Woodall, J. M. Squyres, Open MPI: A flexible high performance MPI, in *Parallel Processing and Applied Mathematics: 6th International Conference, PPAM 2005* (Springer, 2006), pp. 228–239.
